# The etiologies of non-CF bronchiectasis in childhood: a systematic review of 989 subjects

**DOI:** 10.1186/s12887-014-0299-y

**Published:** 2014-12-10

**Authors:** Kelly S Brower, Michael T Del Vecchio, Stephen C Aronoff

**Affiliations:** St. Christopher’s Hospital for Children, Philadelphia, USA; Department of Pediatrics, Temple University School of Medicine, Philadelphia, PA 19140 USA

**Keywords:** Non-CF bronchiectasis, Children, Etiology

## Abstract

**Background:**

While cystic fibrosis (CF) is the most common cause of bronchiectasis in childhood, non-CF bronchiectasis is associated with a wide variety of disorders. The objective of this study was to determine the relative prevalence and specific etiologies on non-CF bronchiectasis in childhood.

**Methods:**

EMBASE, Medline, OVID Cochrane Reviews, Directory of Open Access Journals, Open Science Directory, EPSCO information services, and OAlster were searched electronically and the bibliographies of selected studies were searched manually. The search was conducted independently by 2 authors. Study Selection: (1) any clinical trial, observational study or cross-sectional case series of 10 or more patients with a description of the conditions associated with bronchiectasis; (2) subjects aged 21 years or younger; (3) cystic fibrosis was excluded and; (4) the diagnosis was confirmed by computed tomography of the chest. Data Extraction: Patient number, age range, inclusion criteria, diagnostic criteria, patient source, and categorical and specific etiology.

**Results:**

From 491 studies identified, 12 studies encompassing 989 children with non-CF bronchiectasis were selected. Sixty-three percent of the subjects had an underlying disorder. Infectious (17%), primary immunodeficiency (16%), aspiration (10%), ciliary dyskinesia (9%), congenital malformation (3%), and secondary immunodeficiency (3%) were the most common disease categories; 999 etiologies were identified. Severe pneumonia of bacterial or viral etiology and B cell defects were the most common disorders identified.

**Conclusions:**

The majority of children with non-CF bronchiectasis have an underlying disorder. A focused history and laboratory investigated is recommended.

**Electronic supplementary material:**

The online version of this article (doi:10.1186/s12887-014-0299-y) contains supplementary material, which is available to authorized users.

## Background

Bronchiectasis in children without cystic fibrosis (non-CF bronchiectasis) is believed to be the end result of chronic or repeated episodes of environmental insults superimposed on a background of “genetic vulnerability”; these events lead to bronchial injury and dilatation [1]. In 1963, Clark described 116 cases of bronchiectasis in children aged 0 to 11 years [2]. See of those cases with an apparent etiology, most followed episodes of measles or pertussis. Radiographic evaluation of these children yielded a mixed picture: 1 child had collapse of an entire lung; 12 children had pulmonary cavitation with or without accompanying atelectasis and; 34 children had lobar atelectasis. Bronchography demonstrated bronchiectasis in all subjects tested.

The underlying “vulnerabilities” in children are poorly defined. McDonnell, et al. generated a list of disorders that included immunodeficiency, connective tissue disorders, allergic bronchopulmonary aspergillosis as well as miscellaneous conditions including amyloidosis and endometriosis [3]. In another review, autoimmune disorders, primary ciliary dyskinesia, hypersensitivity syndromes, connective tissue disorders, and malignancy were listed among the potential causes of non-CF bronchiectasis [4]. In both cases, the etiologies listed were not specific for children and were not empirically derived. The goals of this systematic review were to determine the specific etiologies and relative prevalence of these disorders among children with non-CF bronchiectasis from studies reported in the literature.

## Methods

### Protocol

This study followed the Preferred Reporting Items in Systematic Reviews and Meta-Analyses (PRISMA) guidelines [5].

### Eligibility

The study protocol was developed by the authors *a priori*. The inclusion criteria for this review were: (1) any clinical trial, observational study or cross-sectional case series of 10 or more patients that included a delineation of the etiologies and/or the associated conditions with bronchiectasis; (2) subjects aged 21 years or younger; (3) cystic fibrosis was excluded as a diagnosis and; (4) the diagnosis of bronchiectasis was confirmed by computed tomography of the chest. Studies of adults and children were acceptable if the pediatric data was reported separately. Case reports, editorials and review articles were excluded.

### Information sources

EMBASE, Medline, OVID Cochrane Reviews, Directory of Open Access Journals, Open Science Directory, EPSCO information services, and OAlster were searched from 1966 to March 25, 2014. The bibliographies of all of the selected studies were also reviewed.

### Search

The main search term was “non-CF bronchiectasis”. The following filters were used: human, all children and young adult. The searches were performed independently by two of the authors and the results were compared.

### Study selection

Initial evaluation of each article was performed by one author (KSB) and then reviewed by another (MTD). In cases where study populations appeared to overlap, the study with the largest number of subjects was selected. Differences in judgment were resolved first by consensus; ties were adjudicated by the third author (SCA). All studies selected for inclusion were reviewed by the third author.

### Data collection

For each selected study, the following information was recorded: inclusion criteria, number of patients, age range, diagnostic criteria, patient source and country of origin. Categorical and specific etiologies of bronchiectasis were also recorded for each study. Major categories of disease included primary immunodeficiencies, ciliary dyskinesia, infection, aspiration, idiopathic or unknown, congenital malformation, secondary immunodeficiencies, asthma, skeletal disorders, bronchiolitis obliterans, and others. Within each category, specific etiologies were catalogued from those studies that provided specific data.

### Synthesis of results

The categorical and specific etiologies of non-CF bronchiectasis were pooled to provide estimates of the relative prevalence for each disorder. Total sample size estimates for categorical comparisons were calculated from the total number of etiologies identified for the entire review. Total sample size estimates for the comparison of specific etiologies within individual categories were calculated from the total number of etiologies reported in a given category.

### Sources of bias across studies

Patient sampling by number, locale and institution raised the concern of population homogeneity and possible over- or under-representation of a specific area or ethnic group. Variability in diagnostic evaluation and the identification of multiple etiologies for individual patients were also potential sources of bias in defining etiology. Inconsistencies and vagaries in nomenclature were a potential source of error when studies were combined.

## Results and discussion

### Study selection

**Figure 1:**
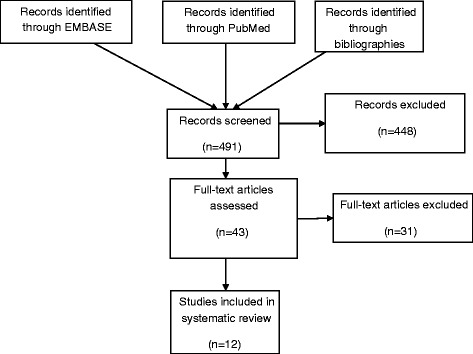
**Results of literature search.**

The results of the literature search are shown in Figure 1. Searches of the Medline and EMBASE databases yielded 202 references. An additional 289 citations were found by extensively searching the bibliographies of selected articles (Additional file 1). No additional studies were found by searching the OVID Cochrane Reviews, Directory of Open Access Journals, Open Science Directory, EPSCO information services, or OAlster. From the 491 studies identified, 448 studies were excluded after a cursory review of the title, abstract, and, when necessary, the results section. The full text of the remaining 43 articles was reviewed in detail. Thirty- one of the remaining studies were excluded: 22 reports had overlapping populations with other studies; 3 did not use computed tomography for the diagnosis of bronchiectasis; 1 did not have the minimum number of patients; 2 studies included the same subjects as previous publications; 2 included adult populations that could not be separated from the pediatric subjects and; 1 did not contain any etiology data.

### Study characteristics and outcomes

The characteristics of the 12 studies that met the inclusion criteria and comprise the basis of this review are shown in Table 1 [6-17]. The reports ranged in size from 22 to 151 participants per study. Non-CF bronchiectasis was defined by computed tomography [18]; in one study, 96% of patients underwent computed tomography [6]. Together, these reports represent a worldwide sample (Australia, Ireland, Turkey, Saudi Arabia, United Kingdom, New Zealand, Alaska, Italy, and Korea). Children were drawn from single centers in 8 studies [6-12,16], multiple centers in 3 studies [13-15] and an entire region in 1 study [17]; the total number of patients included in this review is 989.

**Table 1:** **Summary of Included studies**

**Study citation**	**Inclusion criteria**	**# of patients**	**Age range of patients**	**Diagnostic criteria**	**Patient source**	**Country of origin**
Kapur et. al (2012) [9]	Children < 18 years	113	3-195 months	HRCT scan	Single children’s hospital	Australia
	HRCT scan diagnosis of bronchiectasis					
	Availability of BAL fluid cytology					
	Microbiological results from bronchoscopy					
	No CF diagnosis					
Zaid et. al (2010) [17]	Children < 18 years with discharge diagnosis of chronic bronchitis, bronchiectasis, or chronic suppurative lung disease	92	1.5-13 years	HRCT scan Clinical diagnosis	All Irish public hospitals’ discharge data	Ireland
	Verified with chart review, exclusion of CF and radiology review of HRCT					
	No CF diagnosis (sweat chloride < 60)					
Karakoc et. al (2009) [11]	Diagnosis of bronchiectasis based on suggestive clinical and radiological features confirmed by HRCT	22	87.0 +/− 56.85 months [1]	HRCT scan and clinical diagnosis	Dept of Allergy and Immunology at University Center	Turkey
Banjar (2007) [6]	Non-CF bronchiectasis based on CXR and/or CT chest	151	7.3 +/− 4.1 years	CXR	Single center	Saudi Arabia
				CT chest (96%)		
Li et. al (2005) [13]	Database search bronchiectasis, chronic suppurative lung disease, and chronic cough	136	3.1-18.1 years [2]	HRCT scan	Two centers	United Kingdom
	HRCT diagnosed bronchiectasis with suggestive clinical feature					
	No CF diagnosis (sweat test, genetic mutations, nasal potential differences and faecal elastase if equivocal)					
Karadag et. al (2005) [10]	Patients with non-CF bronchiectasis confirmed with HRCT	111	7.4 +/− 3.7 years	HRCT scan	Single center	Turkey
Eastham et. al (2004) [7]	Children with bronchiectasis confirmed with HRCT	93	1.6-18.8 years	HRCT scan	Single center	United Kingdom
Munro et. al (2011) [14]	All children had HRCT and had at least 5 years of follow up	91	0.9-16 years	HRCT scan	Database of single children’s hospital	New Zealand
	Series of investigations to exclude CF and identify the presumed etiology for bronchiectasis					
Singleton et. al (2000) [16]	Assessed by a pediatric pulmonologist to have definite (CT findings) bronchiectasis [3]	28	1-15 years	HRCT	Two centers	Alaska
Gaillard et. al (2003) [8]	Database search identifying children with 2 or more HRCT scans of the lungs in whom bronchiectasis was reported in the first scan, then reviewed by a single consultant radiologist	22	1-16 years [4]	HRCT	Single children’s hospital	United Kingdom
	Exclusion of CF patients					
Koh et. al (1997) [12]	Clinical features of bronchiectasis conformed by CT and by bronchoscopy when necessary	25	13.1 +/− 2.6 years [2]	Clinical plus CT	Single clinic	Korea
Santamaria et. al (2008) [15]	Bronchiectasis identified by HRCT	105	0-14.4 years	HRCT	Two centers	Italy

The underlying conditions associated with non-CF bronchiectasis in children, by study, are shown in Table 2. Banjar, et al. and Eastham et al. reported multiple associations in individual patients [6,7]. Attempts to contact the authors were unsuccessful. With the inclusion of these studies, the total number of associations (994) exceeds the total number of patients reported (989). In two studies, the most likely association of multiple associations reported for individual patients was the only one counted (e.g. primary immunodeficiency is a more likely association than infection since it was the likely predisposition for the infection) [7,8]. Singleton et al. described patients with definite and probable bronchiectasis [16]; only definite cases were included.

**Table 2:** **Etiology of non-CF Bronchiectasis in childhood by study**

	**Primary immuno- deficiency N/%**	**Ciliary dyskinesia N/%**	**Infection N/%**	**Aspiration N/%**	**Idiopathic N/%**	**Congenital malformation N/%**	**Secondary immuno-deficiency N/%**	**Asthma N/%**	**Skeletal diseases N/%**	**Bronchiolitis obliterans N/%**	**Other N/%**
Kapur et. al [9]	13	2	14	10	62	4	5	-	-	3	-
	11.50%	2%	12%	9%	55%	3.50%	4%			3%	
Zaid et. al [17]	20	8	16	17	29	1	-	-	-	1	-
	22%	9%	17%	18%	32%	1%				1%	
Karakoc et. al [11]	2	1	9	-	6	-	-	4	-	-	-
	9.10%	4.50%	41%		27.20%			18.20%			
Banjar et.al [6].	27	21	6	19	60	11	2	-	10	-	-
	17.31%	13.46%	3.85%	12.18%	38.46%	7.05%	1.28%		6.41%		
Li et. al [13]	40	20	5	25	35	5	6	-	-	-	-
	29.40%	14.70%	3.70%	18.40%	25.70%	3.70%	4.40%				
Karadag et. al [10]	17	7	33	4	42	3	-	5	-	-	-
	15.30%	6.30%	29.70%	3.60%	37.80%	2.70%		4.50%			
Eastham et. al [7]	18	1	28	3	17	7	6	-	-	8	5
	19.35%	1.10%	30.10%	3.20%	18.30%	7.50%	6.45%			8.60%	5.40%
Munro et. al [14]	8	-	21	9	41	-	10	-	1	-	1
	9%		23%	10%	45%		11%		1%		1%
Singleton et.al [16]	-	-	26	2	-	-	-	-	-	-	-
			93%	7%							
Gaillard et.al [8]	4	-	2	2	3	3	-	7	-	-	1
	18.20%		9.10%	9.10%	13.60%	13.60%		31.80%			4.60%
Koh et. al [12]	-	6	6	-	13	-	-	-	-	-	-
		24%	24%		52%						
Santamaria et. al [15]	11	25	7	4	58	-	-	-	-	-	-
	10.5%	23.8%	8.7%	3.8%	55.2%						

### Synthesis of results

The categorical disease processes associated with childhood non-CF bronchiectasis are shown in Table 3. Nine hundred and ninety nine associations were identified in 989 patients. No association was found in 366 subjects (40%). Of the identified associations, the most common were infection (173 subjects, 19%), primary immunodeficiency (160 subjects, 18%), aspiration/foreign body (95 subjects, 10%) and ciliary dyskinesia, including Kartagener’s Syndrome (91 subjects, 10%).

**Table 3:** **Summary of associations with non-CF bronchiectasis of childhood by disease category (989 patients with 994 associations)**

	**Total number**	**% of total**
No association	308	34%
Infectious	174	19%
Primary immunodeficiency	158	17%
Aspiration/foreign body	91	10%
Primary ciliary dyskinesia	66	7%
Congenital malformation	34	4%
Secondary immunodeficiency	29	3%
Asthma	16	2%
Bronchiolitis obliterans	12	1%
Skeletal diseases	11	1%
Others	7	1%

The infections associated with non-CF bronchiectasis are shown in Table 4. Of the 173 patients with an infectious process, 108 (62%) were identified by a specific disease entity. Pneumonia was the most common association (61%) followed by measles (14%), tuberculosis (11%) and pertussis (5%). Varicella, neonatal pneumonia, allergic bronchopulmonary aspergillosis and adenoviral pneumonia were rarely associated with bronchiectasis.

**Table 4:** **Infectious diseases associated with non-CF bronchiectasis of childhood (n = 108)**

	**Total number**	**% of total**
Pneumonia*	66	61%
Measles	15	14%
Tuberculosis	12	11%
Interstitial pneumonia	3	3%
Varicella	3	3%
Neonatal pneumonia**	1	1%
Allergic Bronchopulmonary Aspergillosis (ABPA)	2	2%
Pertussis	5	5%
Adenovirus	1	1%

*Severe viral or bacterial pneumonia.

**Pneumonia at age 6 months or less.

Of the 160 children with bronchiectasis and primary immunodeficiency, 131 (83%) cases were identified by a specific entity (Table 5). B cell disorders accounted for 97 (73.5%) of the primary immunodeficiencies identified: IgG and IgG subclass deficiencies were the most common (66.5%) and IgA deficiency accounted for 6%. A heterogeneous group of combined immunodeficiency disorders accounted for 10% of cases while 7.5% of primary immunodeficiencies resulted from T cell disorders. Of the 29 subjects with secondary immunodeficiencies, 18 (62%) were children who had received chemotherapy for an underlying oncologic process; 6 (20%) of children had HIV/AIDS and 5 (18%) were cardiac transplant recipients.

**Table 5:** **Primary immunodeficiencies associated with non-CF bronchiectasis in childhood (n = 131)**

	**Total number**	**% of total**
**B cell disorders**	**97**	**74%**
IgG deficiency*	63	48%
IgG subclass deficiency	24	18%
IgA deficiency	9	7%
B cell deficiency NOS	1	1%
**T cell disorders**	**9**	**7%**
Hyper IgE syndrome	3	2%
Hyper IgM syndrome	2	2%
T Cell deficiency	3	2%
Chronic mucocutaneous candidiasis	1	1%
**Combined immunodeficiency**	**13**	**10%**
Severe combined immunodeficiency**	9	7%
Ataxia-telangiectasia	2	2%
Wiskott-Aldrich syndrome	2	2%
**Chronic granulomatous disease**	**7**	5%
**Barre lymphocyte syndrome/MHC class II deficiency**	**2**	**2%**
**Mannose-binding protein deficiency**	**1**	**1%**
**Other disorders**	**2**	**2%**

*Includes patients identified as common variable immunodeficiency (30), IgG deficiency (13), agammaglobulinemia (10) and antibody deficiency or dysfunction (10).

**Not otherwise specified.

Of the 95 children with bronchiectasis and aspiration, 18 instances (20%) resulted from aspiration of a foreign body; 14 children (15%) had seizures and recurrent aspiration. Thirty-four children had an underlying congenital malformation and 27 (79%) had a specific entity identified (Table 6). Tracheoesophageal fistulae and cystic lung disease accounted for 52% and 19% of cases, respectively.

**Table 6:** **Congenital malformations associated with non-CF bronchiectasis of childhood**

	**Total number**	**% of total**
Tracheo-oesophageal fistula	14	52%
Cystic lung disease	5	19%
Bronchogenic cyst	2	7%
Yellow nail syndrome	1	4%
Tracheomalacia	1	4%
Congenital lobar emphysema	1	4%
Pulmonary artery sling	1	4%
Bronchial atresia	1	4%
Bronchomalacia	1	4%
**Total**	27	

### Risk of bias

The studies included in this review ranged in size from 22 to 151 subjects; the largest study accounted for 17% of the total sample. The countries of origin contributed samples from 9 different countries and multiple continents. Only 1 study drew patients from a large, regional database; the remaining studies represented one or two clinical sites. All of the studies were retrospective in nature and none employed a standardized diagnostic evaluation. Vagaries in nomenclature occurred among patients diagnosed with primary immunodeficiencies deficiencies. Patients with antibody deficiency, antibody dysfunction or IgG deficiency were grouped together as IgG deficiency; those with combined immunodeficiency were grouped together with severe combined immunodeficiency. Identifying patients with “idiopathic” disease is also confusing since this implies a singular, as yet unidentified process. Three studies reported multiple underlying disorders for individual patients with non-CF bronchiectasis [6-8]. In two, patients with multiple ascribed etiologies could not be identified [6,7].

Non-CF bronchiectasis in children usually has an indolent onset and presents with chronic respiratory symptoms [2-4]. Cough with daily sputum production is the most common clinical presentation and may be present for years before diagnosis. Hemoptysis, pleuritic chest pain, pulmonary osteoarthropathy, and delayed growth are additional findings associated with non-CF bronchiectasis. The definitive diagnosis of bronchiectasis requires chest imaging usually with high resolution computed tomography [18].

While the pathophysiology of bronchiectasis is well defined, the etiologies of non-CF bronchiectasis are varied [1,3]. The data presented in this review suggest that 60% of children with this disorder have an underlying etiology. Infections, primary immunodeficiencies, aspiration (both foreign body and recurrent aspiration in neurodevelopmentally challenged patients) and ciliary dyskinesia account for most cases; severe bacterial or viral pneumonias and IgG deficiencies are the most common etiologies encountered. Throughout the literature, patients without an identified etiology are reported as idiopathic disease. A focused medical history and focused laboratory investigation should reveal the etiology of non-CF bronchiectasis in many cases.

Selection bias is a major limitation of this review. Since all of the studies selected for review were retrospective in design, the possibility exists that patients may have been missed. The subjects included in this study represent 9 different countries, lending credence to the notion that the sample is unbiased. Conversely, U.S. children are underrepresented since the only American study was from Alaska. Finally, 8 of the studies selected for review reported patients from a single clinical site and 3 studies used 2 clinical sites; only 1 study employed a regional database [17].

Misidentification and failure to identify an etiology also contribute to the limitation of this review. Identifying multiple etiologies in individual patients occurred in 2 studies, raising the concern of over representation of specific etiologies [6,7]. The selected studies contained little to no detail regarding the diagnostic approach used to identify the etiology of non-CF bronchiectasis. The absence of a detailed, unified approach to the diagnostic evaluation of these children across studies may have overestimated the number of children without a diagnosis or may have misdiagnosed an unknown number of subjects. Variability in nomenclature compounds this problem. The most common etiology of non-CF bronchiectasis was severe pneumonia, but detail regarding the infectious agent was not available. The second most common etiology was a B cell disorder; unfortunately some subjects were identified with IgG deficiency or an antibody disorder leaving the true diagnosis open to speculation.

## Conclusions

The majority of children with non-CF bronchiectasis have an underlying cause of the disorder. Severe pneumonia, B cell abnormalities, recurrent aspiration or aspiration of a foreign body and ciliary dyskinesia are the most common etiologies. A focused history and laboratory investigation is suggested in the evaluation of these children. A large prospective study with a predefined diagnostic evaluation is required to substantiate the conclusions of this review.

### Ethics

This study did not involve any direct contact with primary patient source documents and as such did not require IRB approval or patient consent.

## Additional file

Additional file 1:
**Search Results.** Description: This file contains the combined results of all of the articles uncovered by the search. It includes duplicates, articles rejected based on review of the abstract, articles rejected after complete review and the articles that make up this systematic review.

## Abbreviations

CF: Cystic fibrosis
PRISMA: Preferred reporting items in systematic reviews and meta-analyses
