# Supplementary material for: The etiologies of non-CF bronchiectasis in childhood: a systematic review of 989 subjects
Source: BMC Pediatr. 2014 Dec 10;14:4. doi: 10.1186/s12887-014-0299-y (PMC4275950; doi:10.1186/s12887-014-0299-y)
Supplement: Additional file 1: — Search Results. Description: This file contains the combined results of all of the articles uncovered by the search. It includes duplicates, articles rejected based on review of the abstract, articles rejected after complete review and the articles that make up this systematic review. [file 12887_2014_299_MOESM1_ESM.doc]

Search Results

1. Chang AB, Bell SC, Byrnes CA, Grimwood K, Holmes PW, King PT, Kolbe J, Landau LI, Maguire G, McDonald MI, Reid DW, Thien FC, Torzillo PJ. Chronic suppurative lung disease and bronchiectasis in children and adults in Australia and New Zealand. Med J Aust 2010;193:356–365.

2. Karadag B, Karakoc F, Ersu R, Kut A, Bakac S, Dagli E. Non- cystic-fibrosis bronchiectasis in children: a persisting problem in developing countries. Respiration 2005;72:233–238.

3. Angrill J, Agusti C, de Celis R, Rano A, Gonzalez J, Sole T, Xaubet A, Rodriguez-Roisin R, Torres A. Bacterial colonisation in patients with bronchiectasis: microbiological pattern and risk factors. Thorax 2002;57:15–19.

4. Angrill J, Agusti C, de Celis R, Filella X, Rano A, Elena M, De La Bellacasa JP, Xaubet A, Torres A. Bronchial inflamma- tion and colonization in patients with clinically stable bronchi- ectasis. Am J Respir Crit Care Med 2001;164:1628–1632.

5. Cole PJ. Inflammation: a two-edged sword-the model of bron- chiectasis. Eur J Respir Dis 1986;147:6–15.

6. Martinez-Garcia MA, Soler-Cataluna JJ, Perpina-Tordera M, Roman-Sanchez P, Soriano J. Factors associated with lung func- tion decline in adult patients with stable non-cystic fibrosis bronchiectasis. Chest 2007;132:1565–1572.

7. Kunst H, Wickremasinghe M, Wells A, Wilson R. Nontubercu- lous mycobacterial disease and Aspergillus-related lung disease in bronchiectasis. Eur Respir J 2006;28:352–357.

8. Edwards EA, Asher MI, Byrnes CA. Pediatric bronchiectasis in the twenty-first century: experience of a tertiary children’s hos- pital in New Zealand. J Paediatr Child Health 2003;39:111–117.

9. Banjar HH. Clinical profile of Saudi children with bronchiecta- sis. Ind J Pediatr 2007;74:149–152.

10. Kahn FW, Jones JM. Diagnosing bacterial respiratory infection by bronchoalveolar lavage. J Infect Dis 1987;155:862–869.

11. Nicolai T. Pediatric bronchoscopy. Pediatr Pulmonol 2001;31:

150–164.

12. Chang AB, Boyce NC, Masters IB, Torzillo PJ, Masel JP. Bron-

choscopic findings in children with non-cystic fibrosis chronic

suppurative lung disease. Thorax 2002;57:935–938.

13. Hare KM, Grimwood K, Leach AJ, Smith-Vaughan H, Torzillo PJ, Morris PS, Chang AB. Respiratory bacterial pathogens in the nasopharynx and lower airways of Australian indigenous

children with bronchiectasis. J Pediatr 2010;157:1001–1005.

14. Kapur N, Masters IB, Chang AB. Exacerbations in non cystic fibrosis bronchiectasis: clinical features and exacerbations. Resp

Med 2009;103:1681–1687.

15. Munro KA, Reed PW, Joyce H, Perry D, Twiss J, Byrnes CA,

Edwards EA. Do New Zealand children with non-cystic fibrosis bronchiectasis show disease progression? Pediatr Pulmonol 2011;46:131–138.

16. Eastham KM, Fall AJ, Mitchell L, Spencer DA. The need to redefine non-cystic fibrosis bronchiectasis in childhood. Thorax 2004;59:324–327.

17. van der Bruggen-Bogaarts BA, Broerse JJ, Lammers JW, van Waes PF, Geleijns J. Radiation exposure in standard and high-resolution chest CT scans. Chest 1995;107:113–115.

18. Chang AB, Faoagali J, Cox NC, Marchant JM, Dean B, Petsky HL, Masters IB. A bronchoscopic scoring system for airway secretions—airway cellularity and microbiological validation. Pediatr Pulmonol 2006;41:887–892.

19. King PT, Holdsworth SR, Freezer NJ, Villanueva E, Holmes PW. Microbiologic follow-up study in adult bronchiectasis. Resp Med 2007;101:1633–1638.

20. Armstrong DS, Grimwood K, Carlin JB, Carzino R, Olinsky A, Phelan PD. Bronchoalveolar lavage or oropharyngeal cultures to identify lower respiratory pathogens in infants with cystic fibrosis. Pediatr Pulmonol 1996;21:267–275.

21. Gernez Y, Tirouvanziam R, Chanez P. Neutrophils in chronic inflammatory airway diseases: can we target them and how? Eur Respir J 2010;35:467–469.

22. Vaneechoutte M, Verschraegen G, Claeys G, Weise B, Van den Abeele AM. Respiratory tract carriers of Moraxella (Branha- mella) catarrhalis in adults and children and interpretation of the isolation of M. catarrhalis from sputum. J Clin Microbiol 1990;28:74–80.

23. Wickremasinghe M, Ozerovitch LJ, Davies G, Wodehouse T, Chadwick MV, Abdallah S, Shah P, Wilson R. Non-tuberculous mycobacteria in patients with bronchiectasis. Thorax 2005;60: 1045–1051.

24. Tunney MM, Field TR, Moriarty TF, Patrick S, Doering G, Muhlebach MS, Wolfgang MC, Boucher R, Gilpin DF, McDowell A, Elborn JS. Detection of anaerobic bacteria in high numbers in sputum from patients with cystic fibrosis. Am J Respir Crit Care Med 2008;177:995–1001.

25. Grimwood K. Airway microbiology and host defences in pediat- ric non-CF bronchiectasis. Paediatr Resp Rev 2011;12:111–118.

26. de Vrankrijker AMM, Wolfs TFW, Ciofu O, Hoiby N, van der

Ent CK, Poulsen SS, Johansen HK. Respiratory syncytial virus infection facilitates acute colonization of Pseudomonas aerugi- nosa in mice. J Med Virol 2009;81:2096–2103.

1. Brown MA, Leman RJ. Bronchiectasis. In: Chernick V, Boat T, Eds. Kendig’s Disorders of respiratory tract in children. 6th Edn. Philadelphia, W.B. Saunders, 1998; pp. 538 560

2. Lewiston NJ. Bronchiectasis in childhood. Pediatr Clin North Am 1984; 31:865 78.

3. Lewitson NJ. Bronchiectasis. In: Hilman B, ed. Pediatric respiratory disease. Philadelphia: WB Saunders, 1993:222

4. Waite DA, Wakefield SJ, Moriarty KM et al. Polynesian bronchiectasis. Eur J Respir Dis Suppl 1983; 127:31 6.

5. Karakoc GB, Yilmaz M, Altintas DU et al. Bronchiectasis: still a problem. Pediatr Pulmonol 2001; 32:175 8.

6. Singleton R, Morris A, Redding G et al. Bronchiectasis in Alaska native children: causes and clinical courses. Pediatr Pulmonol 2000; 29:182 7.

7. Cole P. The damaging role of bacteria in chronic lung infection. J Antimicrob Chemotherapy 1997; 40: 5 10.

8. Hansell DM. Bronchiectasis. Radiol Clin North Am 1998; 36:107 128.

9. Nikolaizik WH, Warner JO. Aetiology of chronic suppurative lung Disease Arch Dis Child 1994; 70:141 2.

10. Eastham KM, Fall AJ, Mitchell L, Spencer DA. The need to redefine non-cystic fibrosis bronchiectasis in childhood. Thorax 2004; 59:324 - 327.

11. Twiss J, Metcalfe R, Edwards E, Byrnes C New Zealand National incidence of bronchiectasis "too high" for a developed Country Arch Dis Child. 2005Jul; 90: 737-40.

12. Sˆ⁄yˆ⁄jˆ⁄kangas O, Keistinen T, Tuuponen TO et al. Evaluation Of the incidence and age distribution of bronchiectasis from The Finish Hospital Discharge Register. Cent Eur J Public Health 1998; 6:235 7.

13. Meeks M, Bush A. Primary ciliary dyskinesia. Pediatric Pulmonology 1999; 29,307-16

14. Bush A, O Callaghan C. Primary ciliary dyskinesia Archives of Disease in Childhood 2002; 87:363-5

15. Bush A, Cole P, Hariri M et al. Primary ciliary dyskinesia: diagnosis and standards of care. Eur Respir J 1998; 12: 982

16. Ramsey BW, Pepe MS, Quan JM et al. Intermittent Administration of inhaled tobramycin in patients with cystic Fibrosis. Cystic Fibrosis Inhaled Tobramycin Study Group. N Engl J Med 1999; 340:23 30.

17. Ellerman A, Bisgaard H. Longitudinal study of lung function in a cohort of primary ciliary dyskinesia. Eur Respir J 1997; 10:2376 9.

18. Li M, Sonnappa S, Lex C, Wong E, Zacharasiewicz A, Bush A, A. Jaffe. Non-CF bronchiectasis: does knowing the aetiology leads to changes in management? Eur. Respir. J., July 1, 2005; 26: 8 - 14.

1. Chang AB, Redding GJ. Bronchiectasis. In: Chernick V, Boat T, Wilmott R, Bush A, editors. Kending’s disorders of respiratory tract in children, 7th edition. Philadelphia: Saunders; 2006. pp. 463 – 477.

2. Dagli E. Noncystic fibrosis bronchiectasis. Pediatr Respir Rev 2000;1:64 – 70.

###3. Barker AF, Bardana EJ. Bronchiectasis: update of an orphan disease. Am Rev Respir Dis 1988;137:969 – 978.

4. Karakoc GB, Yilmaz M, Altintas DU, Kendirli SG. Bronchiec- tasis: still a problem. Pediatr Pulmonol 2001;32:175 – 178.

5. Karadag B, Karakoc F, Ersu R, Kut A, Bakac S, Dagli E. Non- cystic-fibrosis bronchiectasis in children: a persisting problem in developing countries. Respiration 2005;72:233 – 238.

6. Dog ̆ru D, Nik-Ain A, Kiper N, Go ̈c ̧men A, Ozc ̧elik U, Yalc ̧in E, Aslan AT. Bronchiectasis: the consequence of late diagnosis in chronic respiratory symptoms. J Trop Pediatr 2005;51:362– 365.

7. Edwards EA, Asher MI, Byrnes CA. Paediatric bronchiectasis in the twenty-first century: experience of a tertiary children’s hospital in New Zealand. J Paediatr Child Health 2003;39:111– 117.

8. Singleton R, Morris A, Redding G, Poll J, Holck P, Martinez P, Kruse D, Bulkow LR, Petersen KM, Lewis C. Bronchiectasis in Alaska Native children: causes and clinical courses. Pediatr Pulmonol 2000;29:182 – 187.

9. Chang AB, Masel JP, Boyce NC, Wheaton G, Torzillo PJ. Non-CF bronchiectasis: clinical and HRCT evaluation. Pediatr Pulmonol 2003;35:477–483.

10. Steinfort DP, Brady S, Weisinger HS, Einsiedel L. Bronchiectasis in Central Australia: a young face to an old disease. Respir Med 2008;102:574 – 578.

11. Guran T, Ersu R, Karadag B, Akpinar IN, Demirel GY, Hekim N, Dagli E. Association between inflammatory markers in induced sputum and clinical characteristics in children with non-cystic fibrosis bronchiectasis. Pediatr Pulmonol 2007;42:362–369.

12. Eastham KM, Fall AJ, Mitchell L, Spencer DA. The need to redefine non-cystic fibrosis bronchiectasis in childhood. Thorax 2004;59:324 – 327.

13. Angrill J, Agust ́ı C, De Celis R, Filella X, Ran ̃o ́ A, Elena M, De La Bellacasa JP, Xaubet A, Torres A. Bronchial inflammation and colonization in patients with clinically stable bronchiectasis. Am J Respir Crit Care Med 2001;164:1628–1632.

14. Zheng L, Lam WK, Tipoe GL, Shum IH, Yan C, Leung R, Sun J, Ooi GC, Tsang KW. Overexpression of matrix metalloproteinase- 8 and -9 in bronchiectatic airways in vivo. Eur Respir J 2002;20: 170 – 176.

15. Tsang KW, Chan K, Ho P, Zheng L, Ooi GC, Ho JC, Lam W. Sputum elastase in steady-state bronchiectasis. Chest 2000;117: 420 – 426.

16. Stockley RA, Hill SL, Morrison HM, Starkie CM. Elastolytic activity of sputum and its relation to purulence and to lung function in patients with bronchiectasis. Thorax 1984;39:408– 413.

17. Sepper R, Konttinen YT, Ingman T, Sorsa T. Presence, activities, and molecular forms of cathepsin G, elastase, alpha 1-antitrypsin, and alpha 1-antichymotrypsin in bronchiectasis. J Clin Immunol 1995;15:27 – 34.

18. Shum DK, Chan SC, Ip MS. Neutrophil-mediated degradation of lung proteoglycans: stimulation by tumor necrosis factor-alpha in sputum of patients with bronchiectasis. Am J Respir Crit Care Med 2000;162:1925 – 1931.

19. Prikk K, Maisi P, Pirila ̈ E, Sepper R, Salo T, Wahlgren J, Sorsa T. In vivo collagenase-2 (MMP-8) expression by human bronchial epithelial cells and monocytes/macrophages in bronchiectasis. J Pathol 2001;194:232–238.

20. Sepper R, Konttinen YT, Buø L, Eklund KK, Lauhio A, Sorsa T, Tschesche H, Aasen AO, Sillastu H. Potentiative effects of neutral proteinases in an inflamed lung: relationship of neutrophil procollagenase (proMMP-8) to plasmin, cathepsin G and tryptase in bronchiectasis in vivo. Eur Respir J 1997;10:2788 – 2793.

21. Horva ́th I, Hunt J, Barnes PJ, Alving K, Antczak A, Baraldi E, Becher G, van Beurden WJ, Corradi M, Dekhuijzen R, Dweik RA, Dwyer T, Effros R, Erzurum S, Gaston B, Gessner C, Greening A, Ho LP, Hohlfeld J, Jo ̈bsis Q, Laskowski D, Loukides S, Marlin D, Montuschi P, Olin AC, Redington AE, Reinhold P, van Rensen EL, Rubinstein I, Silkoff P, Toren K, Vass G, Vogelberg C, Wirtz H. ATS/ERS Task Force on exhaled breath condensate. exhaled breath condensate: methodological recom- mendations and unresolved questions. Eur Respir J 2005;26:523– 548.

22. Global Initiative for Asthma. Global Strategy for Asthma Management and Prevention. NHLBI/WHO Workshop Report, Publication Number 95-3659. National Institute of Health and National Heart, Lung and Blood Institute, Bethesda, MD, 1995.

23. Conley ME, Notarangelo LD, Etzioni A. Diagnostic criteria for primary immunodeficiencies. Representing PAGID (Pan- American Group for Immunodeficiency) and ESID (European Society for Immunodeficiencies). Clin Immunol 1999;93:190 – 197.

24. American Thoracic Society. Standardization of spirometry, 1994 update. Am J Respir Crit Care Med 1995;152:1107 – 1136.

25. Ooi GC, Khong PL, Chan-Yeung M, Ho JC, Chan PK, Lee JC, Lam WK, Tsang KW. High-resolution CT quantification of bronchiectasis: clinical and functional correlation. Radiology 2002;225:663 – 672.

26. Dohlman AW, Black HR, Royall JA. Expired breath hydrogen peroxide is a marker of acute airway inflammation in pediatric patients with asthma. Am Rev Respir Dis 1993;148:955 – 960.

27. Edwards EA, Metcalfe R, Milne DG, Thompson J, Byrnes CA. Retrospective review of children presenting with non cystic fibrosis bronchiectasis: HRCT features and clinical relationships. Pediatr Pulmonol 2003;36:87 – 93.

28. Tiddens HA. Detecting early structural lung damage in cystic fibrosis. Pediatr Pulmonol 2002;34:228 – 231.

29. Stockley RA. Neutrophils and protease/antiprotease imbalance. Am J Respir Crit Care Med 1999;160:S49 – S52.

30. Sagel SD, Kapsner RK, Osberg I. Induced sputum matrix metalloproteinase-9 correlates with lung function and airway inflammation in children with cystic fibrosis. Pediatr Pulmonol 2005;39:224 – 232.

31. Konstan MW, Berger M. Current understanding of the inflam- matory process in cystic fibrosis: onset and etiology. Pediatr Pulmonol 1997;24:137 – 142

32. Stone PJ, Konstan MW, Berger M, Dorkin HL, Franzblau C, Snider GL. Elastin and collagen degradation products in urine of patients with cystic fibrosis. Am J Respir Crit Care Med 1995; 152:157 – 162.

33. Regamey N, Ochs M, Hilliard TN, Mu ̈hlfeld C, Cornish N, Fleming L, Saglani S, Alton EW, Bush A, Jeffery PK, Davies JC. Increased airway smooth muscle mass in children with asthma, cystic fibrosis, and non-cystic fibrosis bronchiectasis. Am J Respir Crit Care Med 2008;177:837 – 843.

34. Hilliard TN, Regamey N, Shute JK, Nicholson AG, Alton EW, Bush A, Davies JC. Airway remodelling in children with cystic fibrosis. Thorax 2007;62:1074 – 1080.

35. Delacourt C, Le Bourgeois M, D’Ortho MP, Doit C, Scheinmann P, Navarro J, Harf A, Hartmann DJ, Lafuma C. Imbalance between 95kDa type IV collagenase and tissue inhibitor of metalloproteinases in sputum of patients with cystic fibrosis. Am J Respir Crit Care Med 1995;152:765 – 774.

36. Erlewyn-Lajeunesse MD, Hunt LP, Pohunek P, Dobson SJ, Kochhar P, Warner JA, Warner JO. Bronchoalveolar lavage MMP- 9 and TIMP-1 in preschool wheezers and their relationship to persistent wheeze. Pediatr Res 2008;64:194 – 199.

37. Doherty GM, Kamath SV, de Courcey F, Christie SN, Chisakuta A, Lyons JD, Heaney LG, Ennis M, Shields MD. Children with stable asthma have reduced airway matrix metalloproteinase-9 and matrix metalloproteinase-9/tissue inhibitor of metalloprotei- nase-1 ratio. Clin Exp Allergy 2005;35:1168 – 1174.

38. Ratjen F, Hartog CM, Paul K, Wermelt J, Braun J. Matrix metalloproteases in BAL fluid of patients with cystic fibrosis and their modulation by treatment with dornase alpha. Thorax 2002; 57:930 – 934.

1. Eastham KM, Fall AJ, Mitchell L, et al. The need to redefine non-cystic fibrosis

bronchiectasis in childhood. Thorax 2004;59:324–7.

2. King PT, Holdsworth SR, Freezer NJ, et al. Characterisation of the onset and

presenting clinical features of adult bronchiectasis. Respir Med 2006;100:2183–9.

3. Twiss J, Metcalfe R, Edwards E, et al. New Zealand national incidence of

bronchiectasis ‘‘too high’’ for a developed country. Arch Dis Child 2005;90:737–40.

4. Pasteur MC, Helliwell SM, Houghton SJ, et al. An investigation into causative factors in

patients with bronchiectasis. Am J Respir Crit Care Med 2000;162:1277–84.

5. Li AM, Sonnappa S, Lex C, et al. Non-CF bronchiectasis: does knowing the aetiology

lead to changes in management? Eur Respir J 2005;26:8–14.

6. Gaillard EA, Carty H, Heaf D, et al. Reversible bronchial dilatation in children: comparison of serial high-resolution computer tomography scans of the lungs.

Eur J Radiol 2003;47:215–20.

7. Cole TJ, Freeman JV, Preece MA. British 1990 growth reference centiles for weight, height, body mass index and head circumference fitted by maximum penalized likelihood. Stat Med 1998;17:407–29.

8. Miller MR, Hankinson J, Brusasco V, et al. Standardisation of spirometry. Eur Respir J 2005;26:319–38.

9. Stanojevic S, Wade A, Stocks J, et al. Reference ranges for spirometry across all ages: a new approach. Am J Respir Crit Care Med 2008;177:253–60.

10. Edwards EA, Metcalfe R, Milne DG, et al. Retrospective review of children presenting with non cystic fibrosis bronchiectasis: HRCT features and clinical

relationships. Pediatr Pulmonol 2003;36:87–93.

11. Santamaria F, Montella S, Camera L, et al. Lung structure abnormalities, but normal lung function in pediatric bronchiectasis. Chest 2006;130:480–6.

12. Davies G, Wells AU, Doffman S, et al. The effect of Pseudomonas aeruginosa on pulmonary function in patients with bronchiectasis. Eur Respir J 2006;28:974–9.

13. Martinez-Garcia MA, Soler-Cataluna JJ, Perpina-Tordera M, et al. Factors associated with lung function decline in adult patients with stable non-cystic fibrosis bronchiectasis. Chest 2007;132:1565–72.

14. Twiss J, Stewart AW, Byrnes CA. Longitudinal pulmonary function of childhood bronchiectasis and comparison with cystic fibrosis. Thorax 2006;61:414–8.

15. Evans SA, Turner SM, Bosch BJ, et al. Lung function in bronchiectasis: the influence of Pseudomonas aeruginosa. Eur Respir J 1996;9:1601–4.

16. Keistinen T, Saynajakangas O, Tuuponen T, et al. Bronchiectasis: an orphan disease with a poorly-understood prognosis. Eur Respir J 1997;10:2784–7.

17. Edwards EA, Asher MI, Byrnes CA. Paediatric bronchiectasis in the twenty-first century: experience of a tertiary children’s hospital in New Zealand. J Paediatr Child Health 2003;39:111–7.

18. Karadag B, Karakoc F, Ersu R, et al. Non-cystic-fibrosis bronchiectasis in children: a persisting problem in developing countries. Respiration 2005;72:233–8.

19. Lai HC, Kosorok MR, Sondel SA, et al. Growth status in children with cystic fibrosis based on the National Cystic Fibrosis Patient Registry data: evaluation of various criteria used to identify malnutrition. J Pediatr 1998;132:478–85.

20. Milla CE. Association of nutritional status and pulmonary function in children with cystic fibrosis. Curr Opin Pulm Med 2004;10:505–9.

21. Peterson ML, Jacobs DR Jr, Milla CE. Longitudinal changes in growth parameters are correlated with changes in pulmonary function in children with cystic fibrosis. Pediatrics 2003;112:588–92.

22. Verma N, Bush A, Buchdahl R. Is there still a gender gap in cystic fibrosis? Chest 2005;128:2824–34.

1 Santamaria F, Grillo G, Guidi G, Rotondo A, Raia V, de Ritis G, Sarnelli P, Caterino M, Greco L: Cystic fibrosis: when should high- resolution computed tomography of the chest be obtained? Pediatrics 1998;101:908– 913.

2 Sly PD, Soto-Quiros ME, Landau LI, Hudson I, Newton-John H: Factors predisposing to abnormal pulmonary function after adeno- virus type 7 pneumonia. Arch Dis Child 1984;59:935–939.

3 Nikolaizik WH, Warner JO: Aetiology of chronic suppurative lung disease. Arch Dis Child 1994;70:141–142.

4 Johnston ID, Strachan DP, Anderson HR: Ef- fect of pneumonia and whooping cough in childhood on adult lung function. N Engl J Med 1998;338:581–587.

5 Singleton R, Morris A, Redding G, Poll J, Holck P, Martinez P, Kruse D, Bulkow LR, Petersen KM, Lewis C: Bronchiectasis in Alaska Native children: causes and clinical courses. Pediatr Pulmonol 2000; 29: 182– 187.

6 Pasteur MC, Helliwell SM, Houghton SJ, Webb SC, Foweraker JE, Coulden RA, Flow- er CD, Bilton D, Keogan MT: An investiga- tion into causative factors in patients with bronchiectasis. Am J Respir Crit Care Med 2000;162:1277–1284.

7 Kim CK, Chung CY, Kim JS, Kim WS, Park Y, Koh YY: Late abnormal findings on high- resolution computed tomography after My- coplasma pneumonia. Pediatrics 2000;105: 372–378.

8 Karakoc GB, Yilmaz M, Altintas DU, Ken- dirli SG: Bronchiectasis: still a problem. Pe- diatr Pulmonol 2001;32:175–178.

9 Callahan CW, Redding GJ: Bronchiectasis in children: orphan disease or persistent prob- lem? Pediatr Pulmonol 2002;33:492–496.

10 Chang AB, Masel JP, Boyce NC, Wheaton G, Torzillo PJ: Non-CF bronchiectasis: clinical and HRCT evaluation. Pediatr Pulmonol 2003;35:477–483.

Edwards EA, Metcalfe R, Milne DG, Thomp- son J, Byrnes CA: Retrospective review of children presenting with non cystic fibrosis bronchiectasis: HRCT features and clinical relationships. Pediatr Pulmonol 2003;36:87– 93.

Eastham KM, Fall AJ, Mitchell L, Spencer DA: The need to redefine non-cystic fibrosis bronchiectasis in childhood. Thorax 2004; 59:324–327.

Pifferi M, Caramella D, Bulleri A, Baldi S, Peroni D, Pietrobelli A, Boner AL: Pediatric bronchiectasis: correlation of HRCT, venti- lation and perfusion scintigraphy, and pul- monary function testing. Pediatr Pulmonol 2004;38:298–303.

Redding G, Singleton R, Lewis T, Martinez P, Butler J, Stamey D, Bulkow L, Peters H, Gove J, Morray B, Jones C: Early radiograph- ic and clinical features associated with bron- chiectasis in children. Pediatr Pulmonol 2004;37:297–304.

Callahan CW: Bronchiectasis: abated or aborted? Respiration 2005;72:225–226.

Twiss J, Stewart AW, Byrnes CA: Longitudi- nal pulmonary function of childhood bron- chiectasis and comparison with cystic fibro- sis. Thorax 2006;61:414–418.

Guran T, Ersu R, Karadag B, Akpinar IN, Demirel GY, Hekim N, Dagli E: Association between inflammatory markers in induced sputum and clinical characteristics in chil- dren with non-cystic fibrosis bronchiectasis. Pediatr Pulmonol 2007;42:362–369.

Li AM, Sonnappa S, Lex C, Wong E, Zacha- rasiewicz A, Bush A, Jaffe A: Non-CF bron- chiectasis: does knowing the aetiology lead to changes in management? Eur Respir J 2005;26:8–14.

Notarangelo LD, Plebani A, Mazzolari E, Soresina A, Bondioni MP: Genetic causes of bronchiectasis: primary immune deficien- cies and the lung. Respiration 2007;74:264– 275.

Morillas HN, Zariwala M, Knowles MR: Ge- netic causes of bronchiectasis: primary cili- ary dyskinesia. Respiration 2007;74:252– 263.

Panitch HB: Evaluation of recurrent pneu- monia. Pediatr Infect Dis J 2005;24:265– 266.

King PT, Holdsworth SR, Freezer NJ, Villa- nueva E, Holmes PW: Characterisation of the onset and presenting clinical features of adult bronchiectasis. Respir Med 2006;100: 2183–2189.

Barker AF: Bronchiectasis. N Engl J Med 2002;346:1383–1393.

Santamaria F, Montella S, Camera L, Palum- bo C, Greco L, Boner AL: Lung structure ab- normalities, but normal lung function in pediatric bronchiectasis. Chest 2006;130: 480–486.

Montella S, Andreucci MV, Greco L, Barba- rano F, De Stefano S, Brunese L, Santamaria F: Clinical utility of CT in children with per- sistent focal chest abnormality. Eur Respir J 2005;26:751–752.

- Included

Naidich DP, McCauley DI, Khouri NF, Stitik FP, Siegelman SS: Computed tomography of bronchiectasis. J Comput Assist Tomogr 1982;6:437–444.

Kuhn JP: Pulmonary infection; in Silverman FN, Kuhn JP (eds): Essentials of Caffey’s Pe- diatric X-Ray Diagnosis. Chicago, Year Book Medical Publishers, 1990, pp 277–306.

Reiff DB, Wells AU, Carr DH, et al: CT find- ings in bronchiectasis: limited value in dis- tinguishing between idiopathic and specific types. AJR Am J Roentgenol 1995;165:261– 267.

American Thoracic Society. ATS statement: standardization of spirometry, 1994 update. Am J Respir Crit Care Med 1995;152:1107– 1136.

Zapletal A, Samanek M, Paul T: Lung Func- tion in Children and Adolescents. Methods, Reference Values. Basel, Karger, 1987.

31 Koran LM: The reliability of clinical meth- ods, data and judgment. N Engl J Med 1975; 293:642–646.

32 Chmura K, Chan ED, Noone PG, Zariwala M, Winn RA, Knowles MR, Iseman MD, Gardner EM: A middle-aged woman with recurrent respiratory infections. Respiration 2005;72:427–430.

33 Bush A, O’Callaghan C: Primary ciliary dys- kinesia. Arch Dis Child 2002;87:363–365.

34 Upham JW, Lee PT, Holt BJ, Heaton T, Prescott SL, Sharp MJ, Sly PD, Holt PG: De- velopment of interleukin-12-producing ca- pacity throughout childhood. Infect Immun 2002;70:6583–6588.

35 Fall A, Spencer D: Paediatric bronchiectasis in Europe: what now and where next? Paedi- atr Respir Rev 2006;7:268–274.

36 Grafakou O, Moustaki M, Tsolia M, Kava- zarakis E, Mathioudakis J, Fretzayas A, Ni- colaidou P, Karpathios T: Can chest X-ray predict pneumonia severity? Pediatr Pul- monol 2004;38:465–469.

37 Milic-Emili J, Henderson JA, Dolovich MB, Trop D, Kaneko K: Regional distribution of inspired gas in the lung. J Appl Physiol 1966; 21:749–759.

38 Cleveland RH: Symmetry of bronchial an- gles in children. Radiology 1979;133:89–93.

39 Verbanck S, Paiva M: Implication of left-to- right lung ventilation heterogeneity. J Appl Physiol 2000;88:1150–1151.

40 Pulmonary complications of inhalation; in Phelan PD, Landau LI, Olinsky A (eds): Re- spiratory Illness in Children. Oxford, Black- well Scientific Publication, 1982, pp 294– 315.

41 Twiss J, Metcalfe R, Edwards E, Byrnes C: New Zealand national incidence of bronchi- ectasis ‘too high’ for a developed country. Arch Dis Child 2005;90:737–740.

42 Karadag B, Karakoc F, Ersu R, Kut A, Bakac S, Dagli E: Non-cystic-fibrosis bronchiecta- sis in children: a persisting problem in devel- oping countries. Respiration 2005;72:233– 238.

43 Bluestone CD, Stool SE, Alper CM (eds): Pe- diatric Otolaryngology, ed 4. Philadelphia, Saunders and Elsevier Science, 2003.

Mclean AN, Sproule MW, Cowan MD, Thomson NC: High resolution computed to- mography in asthma. Thorax 1998;53:308– 314.

Ip MS, So SY, Lam WK, Yam L, Liong E: High prevalence of asthma in patients with bron- chiectasis in Hong Kong. Eur Respir J 1992; 5:418–423.

Galassi C, De Sario M, Biggeri A, Bisanti L, Chellini E, Ciccone G, Petronio MG, Piffer S, Sestini P, Rusconi F, Viegi G, Forastiere F: Changes in prevalence of asthma and aller- gies among children and adolescents in Italy: 1994–2002. Pediatrics 2006;117:34–42.

Baldacci S, Modena P, Carrozzi L, Pedreschi M, Vellutini M, Biavati P, Simoni M, Sapigni T, Viegi G, Paoletti P, Giuntini C: Skin prick test reactivity to common aeroallergens in relation to total IgE, respiratory symptoms, and smoking in a general population sample of northern Italy. Allergy 1996;51:149–156.

Eigen H, Laughlin JJ, Homrighausen J: Re- current pneumonia in children and its rela- tionship to bronchial hyperreactivity. Pedi- atrics 1982;70:698–704.

1. Callahan CW, Redding G. Bronchiectasis in children: Orphan disease or persistent problem? Pediatr Pulmonol 2002; 33: 492- 496.

2. Karakoc GB, Yilmaz M, Altintas DU, Kendiri SG. Bron- chiectasis: Still a problem. Pediatr Pulmonol 2001; 32: 175-178.

3. Brown MA, Leman RJ. Bronchiectasis. In Chernick V, Boat T, eds. Kendig’s disorder of the respiratory tract in children. 6th edition, Philadelphia; WB Saunders; 1998. 538-560.

4. Biering A. Childhood pneumonia, including pertussis, pneumonia and bronchiectasis: a follow-up study of 151 patients. Acta Pediatr 1956; 45 : 348- 351.

5. Ruberman W, Shaufer I, and Bioondo T. Bronchiectasis and acute pneumonia. Am Rev Tuber 1957; 76: 761-765.

6. Field CE. Bronchiectasis: Third report on a follow-up study of medical and surgical cases from childhood. Arch Dis Child 1969; 44: 551-555

7. Dawson K.P., Bakalinova D. Child bronchiectasis in a desert location. Middle East Pediatrics 1996; 1: 6-8

8. Singleton R, Morris A, Redding G, Poll J, Holck P, Martinez P, Kruse D, Bulkow LR, Peterson KM, Lewis C. Bronchiectasis in Alaska Native children: causes and clinical courses. Pediatr Pulmonol 2000; 29: 182-187.

1 Brown MA, Leman RJ. Bronchiectasis. In: Chernick V, Boat

T, eds. Kendig’s disorders of the respiratory tract in children. 6th Edn. Philadelphia, W.B. Saunders, 1998; pp. 538–560.

2 Singleton R, Morris A, Redding G, et al. Bronchiectasis in Alaska Native children: causes and clinical courses. Pediatr Pulmonol 2000; 29: 182–187.

3 Eastham KM, Fall AJ, Mitchell L, Spencer DA. The need to redefine non-cystic fibrosis bronchiectasis in childhood. Thorax 2004; 59: 324–327.

4 Karakoc GB, Yilmaz M, Altintas DU, Kendiril SG. Bronchiectasis: still a problem. Pediatr Pulmonol 2001; 32: 175–178.

5 Hansell DM. Bronchiectasis. Radiol Clin North Am 1998; 36: 107–128.

6 Brody AS. Cystic fibrosis: when should high-resolution computed tomography of the chest be obtained? Pediatrics 1998; 101: 1071.

7 Reiff DB, Wells AU, Carr DH, Cole PJ, Hansell DM. CT findings in bronchiectasis: limited value in distinguishing between idiopathic and specific types. AJR Am J Roentgenol 1995; 165: 261–267.

8 Lee PH, Carr DH, Rubens MB, Cole PJ, Hansell DM. Accuracy of CT in predicting the cause of bronchiectasis. Clin Radiol 1995; 50: 839–841.

9 Cartier Y, Kavanagh PV, Johkoh T, Mason AC, Muller NL. Bronchiectasis: accuracy of high-resolution CT in the differentiation of specific diseases. AJR Am J Roentgenol 1999; 173: 47–52.

10 Takasugi JE, Godwin JD. The airways. Semin Roentgenol 1991; 26: 175–190.

- Non bronchiectasis related

11 Rosenstein BJ, Cutting GR. The diagnosis of cystic fibrosis: a consensus statement. Cystic fibrosis foundation consen- sus panel. J Pediatr 1998; 132: 589–595.

12 MiddletonPG,GeddesDM,AltonEW.Protocolsforinvivo measurement of the ion transport defects in cystic fibrosis nasal epithelium. Eur Respir J 1994; 7: 2050–2056.

13 Phillips IJ, Rowe DJ, Dewar P, Connett GJ. Faecal elastase 1: a marker of exocrine pancreatic insufficiency in cystic fibrosis. Ann Clin Biochem 1999; 36: 739–742.

14 RutlandJ,ColePJ.Non-invasivesamplingofnasalciliafor measurement of beat frequency and study of ultrastruc- ture. Lancet 1980; 2: 564–565.

15 Narang I, Ersu R, Wilson NM, Bush A. Nitric oxide in chronic airway inflammation in children: diagnostic use and pathophysiological significance. Thorax 2002; 57: 586–589.

16 Karadag B, James AJ, Gultekin E, Wilson NM, Bush A. Nasal and lower airway level of nitric oxide in children with primary ciliary dyskinesia. Eur Respir J 1999; 13: 1402–1405.

17 Standardization of spirometry, 1994 update. American Thoracic Society. Am J Respir Crit Care Med 1995; 152: 1107–1136.

18 Hadfield PJ, Rowe-Jones JM, Bush A, Makay IS. Treatment of otitis media with effusion in children with primary ciliary dyskinesia. Eur Respir J 1997; 10: 2376–2379.

19 Nikolaizik WH, Warner JO. Aetiology of chronic suppura- tive lung disease. Arch Dis Child 1994; 70: 141–142.

20 Edwards EA, Metcalfe R, Milne DG, Thompson J, Byrnes CA. Retrospective review of children pre- senting with non cystic fibrosis bronchiectasis: HRCT features and clinical relationships. Pediatr Pulmonol 2003; 36: 87–93.

21 Glauser E, Cook C, Harris G. Bronchiectasis: a review of 187 cases in children with follow-up pulmonary function studies in 58. Acta Paediatr Scand 1966; Suppl. 165, 1.

22 Strang C. The fate of children with bronchiectasis. Ann Intern Med 1956; 44: 630–656.

23 Drake AJ, Howells RJ, Shield JPH, Prendiville A, Ward PS, Crowne EC. Symptomatic adrenal insufficiency presenting with hypoglycaemia in children with asthma receiving high dose inhaled fluticasone propionate. BMJ 2002; 324: 1081–1083.

24 Coren ME, Meeks M, Morrison I, Buchdahl RM, Bush A. Primary ciliary dyskinesia: age at diagnosis and symptom history. Acta Paediatr 2002; 91: 667–669.

25 Ellerman A, Bisgaard H. Longitudinal study of lung function in a cohort of primary ciliary dyskinesia. Eur Respir J 1997; 10: 2376–2379.

26 Pasteur MC, Helliwell SM, Houghton SJ, et al. An investigation into causative factors in patients with bronchiectasis. Am J Respir Crit Care Med 2000; 162: 1277–1284.

27 Wong-You-Cheong JJ, Leahy BC, Taylor PM, Church SE. Airways obstruction and bronchiectasis: correlation with duration of symptoms and extent of bronchiectasis on computed tomography. Clin Radiol 1992; 45: 256–259.

28 Chang AB, Masel JP, Boyce NC, Wheaton G, Torzillo PJ. Non-CF bronchiectasis: clinical and HRCT evaluation. Pediatr Pulmonol 2003; 35: 477–483.

29 Wilson CB, Jones PW, O’Leary CJ, Hansell DM, Cole PJ, Wilson R. Effect of sputum bacteriology on the quality of life of patients with bronchiectasis. Eur Respir J 1997; 10: 1754–1760.

30 Hernandez C, Abreu J, Jimenez A, Fernandez R, Martin C. Pulmonary function and quality of life in relation to bronchial colonization in adults with bronchiectasis not caused by cystic fibrosis. Med Clin (Barc) 2002; 118: 130–134.

31 Westcott JL. Bronchiectasis. Radiol Clin North Am 1991; 29: 1031–1042.

32 Nadel HR, Stringer DA, Levison H, Turner JA, Sturgess JM. The immotile cilia syndrome: radiological manifestations. Radiology 1985; 154: 651–655.

33 Curtin JJ, Webster ADB, Farrant J, Katz D. Bronchiectasis in hypogammaglobulinaemia – a computed tomography assessment. Clin Radiol 1991; 44: 82–84.

34 Edwards EA, Narang I, Li A, Hansell DM, Rosenthal M, Bush A. HRCT lung abnormalities are not a surrogate for exercise limitation in bronchiectasis. Eur Respir J 2004; 24: 538–544.

1 Dagli E: Non-cystic fibrosis bronchiectasis. Paediatr Respir Rev 2000;1:64–70.

2 Barker AF, Bardana EJ: Bronchiectasis: Up- date of an orphan disease. Am Rev Respir Dis 1988;137:969–978.

3 Nikolaizik WH, Warner JO: Aetiology of chronic suppurative lung disease. Arch Dis Child 1994;70:141–142.

4 Karakoc F, Dagli E, Günay I, Bakac S, Yuksel M, Kiyan G, Dagli T: The outcome and long- term follow-up of children with bronchiectasis. Eur Respir J 1997;10:338.

5 Pasteur MC, Helliwell SM, Houghton SJ, Webb SC, Foweraker JE, Coulden RA, Flower CD, Bilton D, Keogan MT: An investigation into causative factors in patients with bronchi- ectasis. Am J Respir Crit Care Med 2000;162: 1277–1284.

6 Waite DA, Wakefield SJ, Moriaty KM, Lewis ME, Cuttance PC, Scott AG: Polynesian bron- chiectasis. Eur J Respir Dis Suppl 1983;64: 31–36.

7 Singleton R, Morris A, Redding G, Poll J, Hol- ck P, Martinez P, Kruse D, Bulkow LR, Pe- tersen KM, Lewis C: Bronchiectasis in Alaska native children: Causes and clinical courses.

Pediatr Pulmonol 2000;29:182–187.

8 Chang AB, Grimwood K, Mulholland EK, Torzillo PJ: Bronchiectasis in indigenous chil- dren in remote Australian communities. Med J Aust 2002;177:200–204.

9 Field CE: Bronchiectasis: Third report on a fol- low-up study of medical and surgical cases from childhood. Arch Dis Child 1969;44:551– 561.

10 McGuinness G, Naidich DP: CT of airways disease and bronchiectasis. Radiol Clin North Am 2002;40:1–19.

11 McGuinness G, Naidich DP, Leitman BS, et al: Bronchiectasis: CT evaluation. Am J Ra- diol 1993;160:253–259.

American Thoracic Society (ATS) statement: Standardization of spirometry, 1994 update. Am J Respir Crit Care Med 1995;152:1107– 1136.

Knudson RJ, Lebowitz MD, Holberg C, Bur- rows K: Changes in the normal maximal expi- ratory flow-volume curve with growth and ag- ing. Am Rev Respir Dis 1983;127:725–734.

Tsang KW, Ho PL, Lam WK, et al: Inhaled fluticasone reduces sputum inflammatory indi- ces in severe bronchiectasis. Am J Respir Crit Care Med 1998;158:723–727.

Lewinston NJ: Bronchiectasis; in Hilman BC (ed): Pediatric Respiratory Disease. Philadel- phia, Saunders, 1993, pp 222–229.

Ferkol TW, Davis PB: Bronchiectasis and bronchiolitis obliterans; in Taussig LM, Lan- dau LI, Le Souëf PN, Morgan WJ, Martinez FD, Sly PD (eds): Pediatric Respiratory Medi- cine. St Louis, Mosby, 1999, pp 784–789.

Karakoc GB, Yilmaz M, Altintas DU, Ken- dirli SG: Bronchiectasis: Still a problem. Pedi- atr Pulmonol 2001;32:175–178.

Brown MA, Lemen RJ: Bronchiectasis; in Chernick V, Boat TF, Kendig EL (eds): Ken- dig’s Disorders of Respiratory Tract in Chil- dren. Philadelphia, Saunders, 1998, pp 538– 552.

Basaran N, Sayli BS, Basaran A, Solak M, Ar- tan S, Stevenson JD: Consanguineous marria- ges in the Turkish population. Clin Genet 1988;34:339–341.

Hill SL, Mitchell JL, Burnett D, Stockley RA: IgG subclasses in the serum and sputum from patients with bronchiectasis. Thorax 1998;53: 463–468.

Buchdahl RM, Reiser J, Ingram D, Rutman A, Cole PJ, Warner JO: Ciliary abnormalities in respiratory disease. Arch Dis Child 1988;63: 238–243.

22 Kurklu EU, Williams MA, le Roux BT: Bron- chiectasis consequent upon foreign body reten- tion. Thorax 1973;28:601–602.

23 Karakoc F, Karadag B, Akbenlioglu C, Ersu R, Yildizeli B, Yuksel M, Dagli E: Foreign body aspiration: What is the outcome? Pediatr Pul- monol 2002;34:30–36.

24 Barker AF, Couch L, Fiel SB, Gotfried MH, Ilowite J, Meyer KC, O’Donnell A, Sahn SA, Smith LJ, Stewart JO, Abuan T, Tully H, Van Dalfsen J, Wells CD, Quan J: Tobramycin so- lution for inhalation reduces sputum Pseudo- monas aeruginosa density in bronchiectasis. Am J Respir Crit Care Med 2000;162:481–485

25 Tsang KWT, Ho PI, Chan KN, Ip MSM, Lam WK, Ho CS, Yuen KY, Ooi GC, Amitani R, Tanaka E: A pilot study of low-dose erythro- mycin in bronchiectasis. Eur Respir J 1999;13: 361–364.

26 Koh YY, Lee MH, Sun YH, Sung KW, Chae JH: Effect of roxithromycin on airway respon- siveness in children with bronchiectasis: A double-blind, placebo-controlled study. Eur Respir J 1997;10:994–999.

27 Kolbe J, Wells A, Ram FS: Inhaled steroids for bronchiectasis. Cochrane Database Syst Rev 2000;2:CD000996.

28 Callahan CW, Redding GJ: Bronchiectasis in children: Orphan disease or persistent prob- lem? Pediatr Pulmonol 2002;33:492–496.

29 Kutlay H, Cangir AK, Enön S, Sahin E, et al: Surgical treatment in bronchiectasis: Analysis of 166 patients. Eur J Cardiothorac Surg 2002; 21:634–637.

30 Wilson JF, Decker AM: The surgical manage- ment of childhood bronchiectasis. A review of 96 consecutive pulmonary resections in chil- dren with nontuberculous bronchiectasis. Ann Surg 1982;195:354–363.

1 Lewiston NJ. Bronchiectasis in childhood. Pediatr Clin North Am

1984;31:865–78.

2 Lae ̈nnec RTH. A treatise in the diseases of the chest and on mediate

auscultation. 4th edition (1819). Translation by J Forbes. London: Longman,

1834.

3 Waite DA, Wakefield SJ, Moriarty KM, et al. Polynesian bronchiectasis.

Eur J Respir Dis Suppl 1983;127:31–6.

4 Karakoc GB, Yilmaz M, Altintas DU, et al. Bronchiectasis: still a problem.

Pediatr Pulmonol 2001;32:175–8.

5 Singleton R, Morris A, Redding G, et al. Bronchiectasis in Alaska native

children: causes and clinical courses. Pediatr Pulmonol 2000;29:182–7.

6 Clark N. Bronchiectasis in childhood. BMJ 1963;1:80–7.

7 Field C. Bronchiectasis in childhood. III. Prophylaxis, treatment and progress

with a follow-up study of 202 cases of established bronchiectasis. Pediatrics

1949;4:21–46.

8 Lewitson N. Bronchiectasis. In: Hilman B, ed. Pediatric respiratory disease.

Philadelphia: WB Saunders, 1993:222–9.

9 Callahan CW, Redding GJ. Bronchiectasis in children: orphan disease or

persistent problem? Pediatr Pulmonol 2002;33:492–6.

10 Kang EY, Miller RR, Muller NL. Bronchiectasis: comparison of preoperative

thin-section CT and pathologic findings in resected specimens. Radiology

1995;195:649–54.

11 Nikolaizik WH, Warner JO. Aetiology of chronic suppurative lung disease.

Arch Dis Child 1994;70:141–2.

12 van der Bruggen-Bogaarts BA, van der Bruggen HM, van Waes PF, et al.

Screening for bronchiectasis. A comparative study between chest radiography

and high-resolution CT. Chest 1996;109:608–11.

13 Veale D, Rodgers AD, Griffiths CJ, et al. Variability in ciliary beat frequency in

normal subjects and in patients with bronchiectasis. Thorax

1993;48:1018–20.

14 McKenzie S. Cough – but is it asthma? Arch Dis Child 1994;70:1–2.

15 Pasteur MC, Helliwell SM, Houghton SJ, et al. An investigation into causative

factors in patients with bronchiectasis. Am J Respir Crit Care Med

2000;162:1277–84.

16 Ramsey BW, Pepe MS, Quan JM, et al. Intermittent administration of inhaled

tobramycin in patients with cystic fibrosis. Cystic Fibrosis Inhaled Tobramycin

Study Group. N Engl J Med 1999;340:23–30.

17 Ellerman A, Bisgaard H. Longitudinal study of lung function in a cohort of

primary ciliary dyskinesia. Eur Respir J 1997;10:2376–9.

18 Cole P. The damaging role of bacteria in chronic lung infection. J Antimicrob

Chemother 1997;40(Suppl A):5–10.

19 Glauser E, Cook C, Harris G. Bronchiectasis—a review of 187 cases in

children with follow-up pulmonary function studies in 58. Acta Paediatr Scand

1966;165(Suppl).

20 Strang C. The fate of children with bronchiectasis. Ann Intern Med

1956;44:630–56.

21 Castro-Rodriguez JA, Holberg CJ, Wright AL, et al. Association of

radiologically ascertained pneumonia before age 3 yr with asthmalike symptoms and pulmonary function during childhood: a prospective study. Am J Respir Crit Care Med 1999;159:1891–7.

22 Johnston ID, Strachan DP, Anderson HR. Effect of pneumonia and whooping cough in childhood on adult lung function. N Engl J Med 1998;338:581–7.

23 Nicotra MB, Rivera M, Dale AM, et al. Clinical, pathophysiologic, and microbiologic characterization of bronchiectasis in an aging cohort. Chest 1995;108:955–61.

1. Cherniack NS, Carton RW. Factors associated with respiratory insufficiency in bronchiectasis. Am J Med 1966;41:562 – 571.

2. Pande JN, Jain BP, Gupta RG, Guleria JS. Pulmonary ventilation

and gas exchange in bronchiectasis. Thorax 1971;26:727 – 733.

3. Wong-You-Cheong JJ, Leahy BC, Taylor PM, Church SE. Airways obstruction and bronchiectasis: correlation with duration of symptoms and extent of bronchiectasis on computed tomo-graphy. Clin Radiol 1992;45:256 – 259.

4. Cochrane GM, Webber BA, Clarke SW. Effects of sputum on

pulmonary function. Br Med J [Clin Res] 1977;2:1181 – 1183.

5. Ip M, Lauder IP, Wong WY, Lam WK, So SY. Multivariate analysis of factors affecting pulomary function in bronchiectasis.

Respiration 1993;60:45 – 50.

6. Nicotra MB. Bronchiectasis. Semin Respir Infect 1994;9:31 – 40.

7. Currie DC, Cooke JC, Morgan AD, Kerr IH, Delany D, Strickland

B, Cole PJ. Interpretation of bronchograms and chest radiographs in patients with chronic sputum production. Thorax 1987;42: 278 – 284.

8. Silverman PM, Godwin JD. CT/bronchographic correlations in bronchiectasis. J Comput Assist Tomogr 1987;11:52–56.

9. Brody AS. Cystic fibrosis: when should high-resolution computed tomography of the chest be obtained? Pediatrics 1998;101:1071.

10. Hansell DM. Bronchiectasis. Radiol Clin North Am 1998;36:

107 – 128.

11. Lynch DA, Newell J, Hale V, Dyer D, Corkery K, Fox NL, Gerend

P, Fick R. Correlation of CT findings with clinical evaluations in 261 patients with symptomatic bronchiectasis. AJR 1999;173: 53–58.

12. Roberts HR, Wells AU, Milne DG, Rubens MB, Kolbe J, Cole PJ, Hansell DM. Airflow obstruction in bronchiectasis: correlation between computed tomography features and pulmonary function tests. Thorax 2000;55:198 – 204.

13. Sheehan RE, Wells AU, Copley SJ, Desai SR, Howling SJ, Cole PJ, Wilson R, Hansell DM. A comparison of serial computed and functional change in bronchiectasis. Eur Respir J 2002;20:1 – 7.

14. Helbich TH, Heinz-Peer G, Fleischmann D, Wojnarowski C, Wunderbaldinger P, Huber S, Eichler I, Herold CJ. Evolution of CT findings in patients with cystic fibrosis. AJR 1999;173:81 – 88.

15. Shah RM, Sexauer W, Ostrum BJ, Fiel SB, Friedman AC. High- resolution CT in the acute exacerbation of cystic fibrosis: evaluation of acute findings, reversibility of those findings, and clinical correlation. AJR 1997;169:375 – 380.

16. Marchant JM, Masel JP, Dickinson FL, Masters IB, Chang AB.

Application of chest high-resolution computer tomography in young

children with cystic fibrosis. Pediatr Pulmonol 2001;31:24 – 29.

17. Edwards EA, Asher MI, Byrnes CA. Paediatric bronchiectasis in the 21st century: experience of a tertiary children’s hospital in

New Zealand. J Paediatr Child Health 2003;39:111 – 117.

18. Diederich S, Jurriaans E, Flower CD. Interobserver variation in the diagnosis of bronchiectasis on high-resolution computed

tomography. Eur Radiol 1996;6:801 – 806.

19. American Thoracic Society. Standardization of spirometry, 1994

update. Am J Respir Crit Care Med 1995;152:1107 – 1136.

20. Asher MI, Douglas C, Stewart AW, Quinn JP, Hill PM. Lung volumes in Polynesian children. Am Rev Respir Dis 1987;136: 1360-1365.

21. Bhalla M, Turcios N, Aponte V, Jenkins M, Leitman BS, McCauley DI, Naidich DP. Cystic fibrosis: scoring system with thin-section CT. Radiology 1991;179:783–788.

22. Brasfield D, Hicks G, Soong S, Peters J, Tiller R. Evaluation of scoring system of the chest radiograph in cystic fibrosis: a collaborative study. AJR 1980;134:1195–1198.

23. Webb WR, Nuller NL, Naidich DP. High-resolution CT of the lung. Baltimore: Lippincott, Williams & Wilkins; 2001.

24. Singleton R, Morris A, Redding G, Poll J, Holck P, Martinez P, Kruse D, Bulkow LR, Petersen KM, Lewis C. Bronchiectasis in Alaska Native children: causes and clinical courses. Pediatr Pulmonol 2000;29:182 – 187.

25. Karakoc F, Dagli E, Gunay I, Bakac S, Yuksel M, Kiyan G, Dagli TE. The outcome and long-term follow-up of children with bronchiectasis. Eur Respir J 1997;10:171.

26. Karakoc GB, Yilmaz M, Altintas DU, Kendirli SG. Bronchiec- tasis: still a problem. Pediatr Pulmonol 2001;32:175 – 178.

27. Ferkol TW, Davis BD. Bronchiectasis and bronchiolitis obliter- ans. In: Landau T, editor. Pediatric respiratory medicine. St. Louis: Mosby, Inc.; 1999. p 784 – 792.

28. Hinds JR. Bronchiectasis in Maori. N Z Med J 1958;57:328 – 332.

29. Hansell DM, Wells AU, Rubens MB, Cole PJ. Bronchiectasis: functional significance of areas of decreased attenuation at

expiratory CT. Radiology 1994;193:369 – 374.

30. Dakin CJ, Pereira JK, Henry RL, Wang H, Morton JR. Relation-

ship between sputum inflammatory markers, lung function, and lung pathology on high-resolution computed tomography in children with cystic fibrosis. Pediatr Pulmonol 2002;33:475–482.

31. Tiddens HA. Detecting early structural lung damage in cystic fibrosis. Pediatr Pulmonol 2002;34:228 – 231.

32. Robinson T, Leung AN, Northway WH, Blankenberg FG, Chan F, Bloch DA, Holmes TH, Moss RB. Composite CT/PFT score: an outcome measure which markedly improves sensitivity to change in early cystic fibrosis lung disease. Pediatr Pulmonol 2002;24: 298 [abstract].

33. Demirkazik FB, Ariyurek OM, Ozcelik U, Gocmen A, Hassana- bad HK, Kiper N. High resolution CT in chilren with cystic fibrosis: correlation with pulmonary functions and radiographic scores. Eur J Radiol 2001;37:54 – 59.

34. Nasr SZ, Kuhns LR, Brown RA, Hurwitz ME, Sanders GM, Strouse PJ. Use of computerized tomography and chest X-rays in evaluating efficiacy of aerolized recombinant human DNase in cystic fibrosis: a preliminary study. Pediatr Pulmonol 2001;31: 377 – 382.

35. The ALARA (as low as reasonably achievable) Conference. The ALARA concept in pediatric CT intelligent dose reduction. Multi- disciplinary conference organized by the Society of Pediatric Radiology. August 18 – 19, 2001. Pediatr Radiol 2002;32:217 – 313

36. Chang AB, Grimwood K, Mulholland EK, Torzillo PJ. Bronch- iectasis in indigenous children in remote Australian communities. Med J Aust 2002;177:200 – 204.

37. Field CE. Bronchiectasis in childhood. Pediatrics 1949;4:Part I 21–46; Part II 231–248; Part III 355–372.

38. Strang C. The fate of children with bronchiectasis. Ann Intern Med 1956;44:630–656.

39. Clark S. Bronchiectasis in childhood. Br Med J 1963;1:80–88.

40. Glauser EM, Cook CD, Harris GB. Bronchiectasis: a review of 187 cases in children with follow-up pulmonary function studies

in 58. Acta Paediatr Scand 1996;(Suppl 165):1–16.

41. Fleshman JK, Wilson JF, Cohen JJ. Bronchiectasis in Alaska

Native children; 1968. p 517–523.

42. Dagli E. Non cystic fibrosis bronchiectasis. Paediatric respiratory reviews 2000;1:64–70.

1: Kieninger E, Singer F, Tapparel C, Alves MP, Latzin P, Tan HL, Bossley C,

Casaulta C, Bush A, Davies JC, Kaiser L, Regamey N. High rhinovirus burden in

lower airways of children with cystic fibrosis. Chest. 2013 Mar;143(3):782-90.

2: Valery PC, Morris PS, Grimwood K, Torzillo PJ, Byrnes CA, Masters IB, Bauert

PA, McCallum GB, Mobberly C, Chang AB. Azithromycin for Indigenous children with

bronchiectasis: study protocol for a multi-centre randomized controlled trial.

BMC Pediatr. 2012 Aug 14;12:122. PubMed PMID: 22891748; PubMed Central PMCID:

PMC3445847.

3: Olveira G, Olveira C, Dorado A, GarcÃ­a-Fuentes E, Rubio E, Tinahones F,

Soriguer F, Murri M. Cellular and plasma oxidative stress biomarkers are raised

in adults with bronchiectasis. Clin Nutr. 2013 Feb;32(1):112-7. doi:

10.1016/j.clnu.2012.06.002. Epub 2012 Jun 29. PubMed PMID: 22749311.

4: Pizzutto SJ, Grimwood K, Bauert P, Schutz KL, Yerkovich ST, Upham JW, Chang

AB. Bronchoscopy contributes to the clinical management of indigenous children

newly diagnosed with bronchiectasis. Pediatr Pulmonol. 2013 Jan;48(1):67-73. doi:

10.1002/ppul.22544. Epub 2012 Mar 19. PubMed PMID: 22431241.

5: Bothra M, Lodha R, Kabra SK. Tobramycin for the treatment of bacterial

pneumonia in children. Expert Opin Pharmacother. 2012 Mar;13(4):565-71. doi:

10.1517/14656566.2012.656090. Epub 2012 Feb 1. Review. PubMed PMID: 22292783.

6: Hester KL, Macfarlane JG, Tedd H, Jary H, McAlinden P, Rostron L, Small T,

Newton JL, De Soyza A. Fatigue in bronchiectasis. QJM. 2012 Mar;105(3):235-40.

doi: 10.1093/qjmed/hcr184. Epub 2011 Oct 20. PubMed PMID: 22016379.

7: Montella S, Maglione M, Bruzzese D, Mollica C, Pignata C, Aloj G, Manna A,

Esposito A, Mirra V, Santamaria F. Magnetic resonance imaging is an accurate and

reliable method to evaluate non-cystic fibrosis paediatric lung disease.

Respirology. 2012 Jan;17(1):87-91. doi: 10.1111/j.1440-1843.2011.02067.x. PubMed

PMID: 21943039.

8: Kapur N, Grimwood K, Masters IB, Morris PS, Chang AB. Lower airway

microbiology and cellularity in children with newly diagnosed non-CF

bronchiectasis. Pediatr Pulmonol. 2012 Mar;47(3):300-7. doi: 10.1002/ppul.21550.

Epub 2011 Sep 7. PubMed PMID: 21901858.

9: Kapur N, Masters IB, Newcombe P, Chang AB. The burden of disease in pediatric

non-cystic fibrosis bronchiectasis. Chest. 2012 Apr;141(4):1018-24. doi:

10.1378/chest.11-0679. Epub 2011 Sep 1. PubMed PMID: 21885727.

10: Kapur N, Masters IB, Morris PS, Galligan J, Ware R, Chang AB. Defining

pulmonary exacerbation in children with non-cystic fibrosis bronchiectasis.

Pediatr Pulmonol. 2012 Jan;47(1):68-75. doi: 10.1002/ppul.21518. Epub 2011 Aug 9.

PubMed PMID: 21830316.

11: MartÃ­nez-GarcÃ­a MÃ, Soler-CataluÃ±a JJ, CatalÃ¡n-Serra P, RomÃ¡n-SÃ¡nchez P,

Tordera MP. Clinical efficacy and safety of budesonide-formoterol in non-cystic

fibrosis bronchiectasis. Chest. 2012 Feb;141(2):461-8. doi:

10.1378/chest.11-0180. Epub 2011 Jul 21. PubMed PMID: 21778259.

12: Mateos-Corral D, Coombs R, Grasemann H, Ratjen F, Dell SD. Diagnostic value

of nasal nitric oxide measured with non-velum closure techniques for children

with primary ciliary dyskinesia. J Pediatr. 2011 Sep;159(3):420-4. doi:

10.1016/j.jpeds.2011.03.007. Epub 2011 Apr 22. PubMed PMID: 21514598.

13: Tan HL, Regamey N, Brown S, Bush A, Lloyd CM, Davies JC. The Th17 pathway in

cystic fibrosis lung disease. Am J Respir Crit Care Med. 2011 Jul

15;184(2):252-8. doi: 10.1164/rccm.201102-0236OC. Epub 2011 Apr 7. PubMed PMID:

14: Zaid AA, Elnazir B, Greally P. A decade of non-cystic fibrosis bronchiectasis

1996-2006. Ir Med J. 2010 Mar;103(3):77-9. PubMed PMID: 20666070.

15: Pasteur MC, Bilton D, Hill AT; British Thoracic Society Bronchiectasis non-CF

Guideline Group. British Thoracic Society guideline for non-CF bronchiectasis.

Thorax. 2010 Jul;65 Suppl 1:i1-58. doi: 10.1136/thx.2010.136119. Review. PubMed

PMID: 20627931.

16: Pasteur MC, Bilton D, Hill AT; British Thoracic Society Non-CF Bronchiectasis

Guideline Group. British Thoracic Society guideline for non-CF bronchiectasis.

Thorax. 2010 Jul;65(7):577. doi: 10.1136/thx.2010.142778. PubMed PMID: 20627912.

17: Hayes D Jr, Meyer KC. Lung transplantation for advanced bronchiectasis. Semin

Respir Crit Care Med. 2010 Apr;31(2):123-38. doi: 10.1055/s-0030-1249109. Epub

2010 Mar 30. PubMed PMID: 20354926.

18: Kapur N, Masters IB, Chang AB. Longitudinal growth and lung function in

pediatric non-cystic fibrosis bronchiectasis: what influences lung function

stability? Chest. 2010 Jul;138(1):158-64. doi: 10.1378/chest.09-2932. Epub 2010

Feb 19. PubMed PMID: 20173055.

19: YalÃ§in E, Talim B, OzÃ§elik U, DoÄŸru D, CobanoÄŸlu N, Pekcan S, Kiper N. Does

defective apoptosis play a role in cystic fibrosis lung disease? Arch Med Res.

2009 Oct;40(7):561-4. doi: 10.1016/j.arcmed.2009.07.005. Epub 2009 Sep 25. PubMed

PMID: 20082869.

20: Karakoc GB, Inal A, Yilmaz M, Altintas DU, Kendirli SG. Exhaled breath

condensate MMP-9 levels in children with bronchiectasis. Pediatr Pulmonol. 2009

Oct;44(10):1010-6. doi: 10.1002/ppul.21096. PubMed PMID: 19725099.

21: Livnat G, Bentur L, Kuzmisnsky E, Nagler RM. Salivary profile and oxidative

stress in children and adolescents with cystic fibrosis. J Oral Pathol Med. 2010

Jan;39(1):16-21. doi: 10.1111/j.1600-0714.2009.00813.x. Epub 2009 Jul 27. PubMed

PMID: 19656266.

22: Montella S, Santamaria F, Salvatore M, Pignata C, Maglione M, Iacotucci P,

Mollica C. Assessment of chest high-field magnetic resonance imaging in children

and young adults with noncystic fibrosis chronic lung disease: comparison to

high-resolution computed tomography and correlation with pulmonary function.

Invest Radiol. 2009 Sep;44(9):532-8. doi: 10.1097/RLI.0b013e3181b4c1ba. PubMed

PMID: 19652613.

23: Kapur N, Masters IB, Chang AB. Exacerbations in noncystic fibrosis

bronchiectasis: Clinical features and investigations. Respir Med. 2009

Nov;103(11):1681-7. doi: 10.1016/j.rmed.2009.05.007. Epub 2009 Jun 6. PubMed

PMID: 19501498.

24: Voglis S, Quinn K, Tullis E, Liu M, Henriques M, Zubrinich C, PeÃ±uelas O,

Chan H, Silverman F, Cherepanov V, Orzech N, Khine AA, Cantin A, Slutsky AS,

Downey GP, Zhang H. Human neutrophil peptides and phagocytic deficiency in

bronchiectatic lungs. Am J Respir Crit Care Med. 2009 Jul 15;180(2):159-66. doi:

10.1164/rccm.200808-1250OC. Epub 2009 Apr 30. PubMed PMID: 19406984; PubMed

Central PMCID: PMC2714819.

25: Stevens D, Oades PJ, Armstrong N, Williams CA. Early oxygen uptake recovery

following exercise testing in children with chronic chest diseases. Pediatr

Pulmonol. 2009 May;44(5):480-8. doi: 10.1002/ppul.21024. PubMed PMID: 19382220.

26: Rosenthal M, Narang I, Edwards L, Bush A. Non-invasive assessment of exercise

performance in children with cystic fibrosis (CF) and non-cystic fibrosis

bronchiectasis: is there a CF specific muscle defect? Pediatr Pulmonol. 2009

Mar;44(3):222-30. doi: 10.1002/ppul.20899. PubMed PMID: 19206180.

27: Gillham MI, Sundaram S, Laughton CR, Haworth CS, Bilton D, Foweraker JE.

Variable antibiotic susceptibility in populations of Pseudomonas aeruginosa

infecting patients with bronchiectasis. J Antimicrob Chemother. 2009

Apr;63(4):728-32. doi: 10.1093/jac/dkp007. Epub 2009 Feb 4. PubMed PMID:

19193658.

28: Guran T, Ersu R, Karadag B, Karakoc F, Demirel GY, Hekim N, Dagli E.

Withdrawal of inhaled steroids in children with non-cystic fibrosis

bronchiectasis. J Clin Pharm Ther. 2008 Dec;33(6):603-11. doi:

10.1111/j.1365-2710.2008.00951.x. PubMed PMID: 19138237.

29: Redding GJ. Bronchiectasis in children. Pediatr Clin North Am. 2009

Feb;56(1):157-71, xi. doi: 10.1016/j.pcl.2008.10.014. Review. PubMed PMID:

19135586.

30: Bastardo CM, Sonnappa S, Stanojevic S, Navarro A, Lopez PM, Jaffe A, Bush A.

Non-cystic fibrosis bronchiectasis in childhood: longitudinal growth and lung

function. Thorax. 2009 Mar;64(3):246-51. doi: 10.1136/thx.2008.100958. Epub 2008

Dec 3. PubMed PMID: 19052050.

31: Santamaria F, Montella S, Pifferi M, Ragazzo V, De Stefano S, De Paulis N,

Maglione M, Boner AL. A descriptive study of non-cystic fibrosis bronchiectasis

in a pediatric population from central and southern Italy. Respiration.

2009;77(2):160-5. doi: 10.1159/000137510. Epub 2008 Jun 4. PubMed PMID: 18523381.

32: Chang AB, Redding GJ, Everard ML. Chronic wet cough: Protracted bronchitis,

chronic suppurative lung disease and bronchiectasis. Pediatr Pulmonol. 2008

Jun;43(6):519-31. doi: 10.1002/ppul.20821. Review. PubMed PMID: 18435475.

33: Regamey N, Ochs M, Hilliard TN, MÃ¼hlfeld C, Cornish N, Fleming L, Saglani S,

Alton EW, Bush A, Jeffery PK, Davies JC. Increased airway smooth muscle mass in

children with asthma, cystic fibrosis, and non-cystic fibrosis bronchiectasis. Am

J Respir Crit Care Med. 2008 Apr 15;177(8):837-43. doi:

10.1164/rccm.200707-977OC. Epub 2008 Jan 24. PubMed PMID: 18218992.

34: Guran T, Turan S, Karadag B, Ersu R, Karakoc F, Bereket A, Dagli E. Bone

mineral density in children with non-cystic fibrosis bronchiectasis. Respiration.

2008;75(4):432-6. Epub 2007 Jul 6. PubMed PMID: 17622756.

35: Guran T, Ersu R, Karadag B, Akpinar IN, Demirel GY, Hekim N, Dagli E.

Association between inflammatory markers in induced sputum and clinical

characteristics in children with non-cystic fibrosis bronchiectasis. Pediatr

Pulmonol. 2007 Apr;42(4):362-9. PubMed PMID: 17351928.

36: Banjar HH. Clinical profile of Saudi children with bronchiectasis. Indian J

Pediatr. 2007 Feb;74(2):149-52. PubMed PMID: 17337827.

37: Bilton D, Henig N, Morrissey B, Gotfried M. Addition of inhaled tobramycin to

ciprofloxacin for acute exacerbations of Pseudomonas aeruginosa infection in

adult bronchiectasis. Chest. 2006 Nov;130(5):1503-10. PubMed PMID: 17099030.

38: D'Eufemia P, Finocchiaro R, Celli M, Tote J, Ferrucci V, Zambrano A, Troiani

P, Quattrucci S. Neutrophil glutamine deficiency in relation to genotype in

children with cystic fibrosis. Pediatr Res. 2006 Jan;59(1):13-6. Epub 2005 Dec 2.

PubMed PMID: 16327011.

39: Li AM, Sonnappa S, Lex C, Wong E, Zacharasiewicz A, Bush A, Jaffe A. Non-CF

bronchiectasis: does knowing the aetiology lead to changes in management? Eur

Respir J. 2005 Jul;26(1):8-14. PubMed PMID: 15994383.

40: Spencer DA. From hemp seed and porcupine quill to HRCT: advances in the

diagnosis and epidemiology of bronchiectasis. Arch Dis Child. 2005

Jul;90(7):712-4. Review. PubMed PMID: 15970614; PubMed Central PMCID: PMC1720502.

41: Karadag B, Karakoc F, Ersu R, Kut A, Bakac S, Dagli E. Non-cystic-fibrosis

bronchiectasis in children: a persisting problem in developing countries.

Respiration. 2005 May-Jun;72(3):233-8. PubMed PMID: 15942290.

42: Edwards EA, Narang I, Li A, Hansell DM, Rosenthal M, Bush A. HRCT lung

abnormalities are not a surrogate for exercise limitation in bronchiectasis. Eur

Respir J. 2004 Oct;24(4):538-44. PubMed PMID: 15459130.

43: Eastham KM, Fall AJ, Mitchell L, Spencer DA. The need to redefine non-cystic

fibrosis bronchiectasis in childhood. Thorax. 2004 Apr;59(4):324-7. PubMed PMID:

15047953; PubMed Central PMCID: PMC1763810.

44: Robinson P, Carzino R, Armstrong D, Olinsky A. Pseudomonas cross-infection

from cystic fibrosis patients to non-cystic fibrosis patients: implications for

inpatient care of respiratory patients. J Clin Microbiol. 2003 Dec;41(12):5741.

PubMed PMID: 14662972; PubMed Central PMCID: PMC309033.

45: Edwards EA, Metcalfe R, Milne DG, Thompson J, Byrnes CA. Retrospective review

of children presenting with non cystic fibrosis bronchiectasis: HRCT features and

clinical relationships. Pediatr Pulmonol. 2003 Aug;36(2):87-93. PubMed PMID:

12833486.

46: Chang AB, Masel JP, Boyce NC, Wheaton G, Torzillo PJ. Non-CF bronchiectasis:

clinical and HRCT evaluation. Pediatr Pulmonol. 2003 Jun;35(6):477-83. PubMed

PMID: 12746947.

47: Narang I, Ersu R, Wilson NM, Bush A. Nitric oxide in chronic airway

inflammation in children: diagnostic use and pathophysiological significance.

Thorax. 2002 Jul;57(7):586-9. PubMed PMID: 12096200; PubMed Central PMCID:

PMC1746369.

48: Coakley RJ, Taggart C, Canny G, Greally P, O'Neill SJ, McElvaney NG. Altered

intracellular pH regulation in neutrophils from patients with cystic fibrosis. Am

J Physiol Lung Cell Mol Physiol. 2000 Jul;279(1):L66-74. PubMed PMID: 10893204.

49: Phillips IJ, Rowe DJ, Dewar P, Connett GJ. Faecal elastase 1: a marker of

exocrine pancreatic insufficiency in cystic fibrosis. Ann Clin Biochem. 1999

Nov;36 ( Pt 6):739-42. PubMed PMID: 10586310.

50: Osika E, Cavaillon JM, Chadelat K, Boule M, Fitting C, Tournier G, Clement A.

Distinct sputum cytokine profiles in cystic fibrosis and other chronic

inflammatory airway disease. Eur Respir J. 1999 Aug;14(2):339-46. PubMed PMID:

10515411.

51: Russell KJ, McRedmond J, Mukherji N, Costello C, Keatings V, Linnane S, Henry

M, Fitzgerald MX, O'Connor CM. Neutrophil adhesion molecule surface expression

and responsiveness in cystic fibrosis. Am J Respir Crit Care Med. 1998 Mar;157(3

Pt 1):756-61. PubMed PMID: 9517587.

52: Kerem E, Rave-Harel N, Augarten A, Madgar I, Nissim-Rafinia M, Yahav Y,

Goshen R, Bentur L, Rivlin J, Aviram M, Genem A, Chiba-Falek O, Kraemer MR, Simon

Kapur N, Grimwood K, Masters IB, Morris PS, Chang AB. Lower airway microbiology and cellularity in children with newly diagnosed non-CF bronchiectasis. Pediatr Pulmonol 2012;47:300-7.

Eastham KM, Fall AJ, Mitchell L, DA S. The need to redefine non-cystic fibrosis bronchiectasis in childhood. Thorax 2004;59:324-7.

Singleton R MA, Redding G, Poll J, et al. Bronchiectasis in Alaska Native children: causes and clinical courses. Pediatr Pulmonol 2000;29:182-7.

Koh YY, Lee MH, Sun YH, Sung KW, JH C. Effect of roxithromycin on airway responsiveness in children with bronchiectasis: A double-blind, placebo-controlled study. Eur Respir J 1997;10.

A, Branski D, Kerem B. A cystic fibrosis transmembrane conductance regulator

splice variant with partial penetrance associated with variable cystic fibrosis

presentations. Am J Respir Crit Care Med. 1997 Jun;155(6):1914-20. PubMed PMID:

9196095.

1. Lee, J.-K.; Lee, J.; Park, Y.S.; Lee, C.H.; Lee, S.-M.; Yim, J.-J.; Yoo,C.-G.; Kim, Y.W.; Han, S.K. Effect of inhalers on the development of haemoptysis in patients with**non**-**cystic fibrosis bronchiectasis**

International Journal of Tuberculosis and Lung Disease, (1 Mar 2014) Vol.18, No. 3, pp. 363-370

2. Goeminne, Pieter C. Decraene, Ann; Dupont, Lieven J. The Sputum Colour Chart as a predictor of lung inflammation, proteolysis

and damage in **non**-**cystic fibrosis bronchiectasis**: A case-control analysis.Respirology, (February 2014) Vol. 19, No. 2, pp. 203-210.

3. Chalmers, James D.; Finch, Simon. Sputum colour in **non**-**CF bronchiectasis**: The original neutrophil biomarker. Respirology, (February 2014) Vol. 19, No. 2, pp. 153-154.

4. Wilson, Robert; Loebinger, Michael R. Definition and aetiology of **non**-**CF bronchiectasis**. European Respiratory Monograph, (2013) Vol. 60, pp. 107-119.

5. Rademacher, Jessica Ringshausen, Felix C. Prevention and treatment of exacerbations of **non**-**CF bronchiectasis**. European Respiratory Monograph, (2013) Vol. 60, pp. 127-136

6. Subotic, Dragan. Surgical treatment of **bronchiectasis**. European Respiratory Monograph, (2013) Vol. 61, No. 1, pp. 90-106.

7. Trujillano Ruiz, A; Antequera Lardon, T.; Rentero

Redondo, L.; Alonso Herreros, J.M. Efficacy of treatment with inhaled colistin in patients with**bronchiectasis** colonized by Pseudomonas aeruginosa **non cystic fibrosis**. International Journal of Clinical Pharmacy, (December 2013) Vol. 35, No.

6, pp. 1252-1253

8. Ruchaud-Sparagano, Marie-Helene; Gertig, Helen; Macfarlane, James G.;Corris, Paul A.; Simpson, A. John (correspondence); De Soyza, Anthony. Effect of granulocyte-macrophage colony-stimulating factor on neutrophilfunction in idiopathic **bronchiectasis.** Respirology, (November 2013) Vol. 18, No. 8, pp. 1230-1235.

9. Aguirre Zubia, I.; Lizeaga Cundin, G.; Gayan Lera, M.J.;Leunda Eizmendi, L.Inhaled antibiotics usage in exacerbations prophylaxis in patients with**non**-**cystic fibrosis bronchiectasis** International Journal of Clinical Pharmacy, (October 2013) Vol. 35, No. 5,

Supp. SUPPL. 2, pp. 952.

10. Mackley, Rachel. Azithromycin for prevention of exacerbations in **non**-**cystic fibrosis bronchiectasis**. Thorax, (September 2013) Vol. 68, No. 9, pp. 866.

11. Sunny, Syba Susan; Davison, John; De Soyza, Anthony. Management of **non**-**cystic fibrosis bronchiectasis**. Clinical Practice, (September 2013) Vol. 10, No. 5, pp. 629-640.

12. Palladino, A. ;Di Capua, L.; Ruggiero, G.; Spadaro, G.;Genovese, A.

IFN-γ deficiency in adult **bronchiectasis**: A case report. Allergy: European Journal of Allergy and Clinical Immunology, (September

2013) Vol. 68, Supp. SUPPL. 97, pp. 506.

13. El Qarn, Atef Farouk. Alpha 1 antitrypsin deficiency in **non cystic fibrosis bronchiectasis**. Egyptian Journal of Chest Diseases and Tuberculosis, (April 2013) Vol. 62,

No. 2, pp. 311-317

14. Didier, A. [**Non**- **cystic fibrosis bronchiectasis**: Clinical update].

Dilatations des bronches (hors mucoviscidose) : Actualites. Revue des Maladies Respiratoires Actualites, (October 2013) Vol. 5, No. 4,

pp. 247-250.

15. Faria Junior, N. Sleep study on patients with **non**-**cystic fibrosis bronchiectasis**: Apilot study. Sleep Medicine, (December 2013) Vol. 14, Supp. SUPPL. 1, pp. e224.

16. Benan, Musellim; Isil, Uzel; Sermin, Borekci; Bulent,Tutluoglu; Nigar, Halis; Muzeyyen, Erk. Bronchial hyperreactivity in **non**-**cystic fibrosis bronchiectasis**]. Journal of Clinical and Analytical Medicine, (May 2013) Vol. 4, No. 3, pp.224-227.

17. Spinou, Arietta. Physiotherapy assessment of patients with **non**-**cystic fibrosisbronchiectasis**: Current clinical practice and ongoing research. Epitheorese Klinikes Farmakologias kai Farmakokinetikes, (2013) Vol. 31,No. 3, pp. 220-226.

18. Joish, V.N. (correspondence); Spilsbury-Cantalupo, M. Direct medical costs associated with exacerbations related to

**non**-**cystic fibrosis bronchiectasis**. Value in Health, (May 2013) Vol. 16, No. 3, pp. A188.

19. Davison, J. (correspondence); Johnson, G.; Jiwa, K.; Cranson, A.; Wake, R.; Hood, B.; Small, T. Deeper phenotyping of **non CF bronchiectasis** through sputumdifferential counts Thorax, (December 2013) Vol. 68, Supp. SUPPL. 3, pp. A125

20. Salih, W.S. (correspondence); Stretton, R.S.; Fardon, T.C.F.; Chalmers,J.D.C. Does previous exacerbation history predict future exacerbations in**non**-**CF bronchiectasis**?. Thorax, (December 2013) Vol. 68, Supp. SUPPL. 3, pp. A124.

21. Finney, L.J. (correspondence); Beasley, V.; Wan, T.; Cahill, H.; Berry, M.Do specialist **non**-**CF bronchiectasis** clinics improve quality of care?. Thorax, (December 2013) Vol. 68, Supp. SUPPL. 3, pp. A123-A124.

22. Mitchelmore, P. (correspondence); Sheldon, C.; Withers, N. Outpatient survey of patient experience of hypertonic saline use in

**non**-**cystic fibrosis bronchiectasis**. Thorax, (December 2013) Vol. 68, Supp. SUPPL. 3, pp. A122.

23. Bentley, K. (correspondence); Borrill, Z.L. Nebulised hypertonic saline improves quality of life in adult patientswith **non**-**cystic fibrosis bronchiectasis**. Thorax, (December 2013) Vol. 68, Supp. SUPPL. 3, pp. A121-A122.

24. Milosevic, Katarina; Nestorovic, Branimir. Analysis of CFTR gene variants in idiopathic **bronchiectasis** in Serbianchildren. Pediatric, Allergy, Immunology, and Pulmonology, (1 Jun 2013) Vol. 26, No.

2, pp. 93-98.

25. Mckay, C. (correspondence); Pizzutto, S.; Mccallum, G.; Versteegh, L.;Chang, A.B. Clinical profile of children with **non**-**CF bronchiectasis** undergoingbronchoscopy and HRCT in the northern territory. Respirology, (April 2013) Vol. 18, Supp. SUPPL. 2, pp. 75.

26. De Soyza, A. Molecular epidemiological analysis suggests cross infection withpseudomonas aeruginosa is rare in **non**-**cystic fibrosis bronchiectasis**. Thorax, (December 2013) Vol. 68, Supp. SUPPL. 3, pp. A65.

27. Murris-Espin, M. [Pseudomonas aeruginosa and cystic fibrosis and **non**-**cystic fibrosisbronchiectasis** in adults: From colonization to infection]. Revue des Maladies Respiratoires Actualites, (2013) Vol. 5, No. 1, pp.47-53.

28. Lee, A.L. (correspondence); Burge, A.T.; Stirling, R.G.; Holland, A.E. Minimal important difference in six-minute walk distance and incrementalshuttle walk distance in **NON**-**CF bronchiectasis**. Respirology, (April 2013) Vol. 18, Supp. SUPPL. 2, pp. 18.

29. Hwang, Yong Il (correspondence); Kim, Jee Hee; Park, Sunghoon; Jang, SeungHun; Park, Yong Bum; Kim, Dong-Gyu; Hyun, In-Gyu; Lee, Myung-Goo; Jung,Ji-Suck. Hospital admission rate of the patients with **noncystic fibrosisbronchiectasis** during longterm follow up. Chest, (October 2013) Vol. 144, No. 4, Supp.

30. Murray, Maeve P., Dr. (correspondence); Hill, Adam T. Randomized controlled trial of nebulized gentamicin in **non**-**cysticfibrosis bronchiectasis** . . . without patient blinding: Reply. American Journal of Respiratory and Critical Care Medicine, (1 Sep 2012)

Vol. 186, No. 5, pp. 461-462.

31. Harrison, M.J. (correspondence); Murphy, D.M.; Henry, M.T.; Plant, B.J.;Kennedy, M.P. The clinical utility of a hand-held nasal nitric oxide (NNO)

electrochemical analyser to screen patients with **bronchiectasis** for

primary ciliary dyskinesia (PCD) and cystic fibrosis (CF). Irish Journal of Medical Science, (November 2012) Vol. 181, Supp. SUPPL.

10, pp. S403-S404.

32. Ahmed, M. (correspondence); Sharma, K.; O'Regan, A.; O'Mahony, M.; Breen,D.; Gilmartin, J.J.; Rutherford, R. Characteristics of patients with middle lobe/ lingula predominant**bronchiectasis**. Irish Journal of Medical Science, (November 2012) Vol. 181, Supp. SUPPL.

10, pp. S401-S402.

33. Catherinot, E. [News on **bronchiectasis** (**excluding cystic fibrosis**)]. Revue des Maladies Respiratoires Actualites, (October 2012) Vol. 4, No. 5,pp. 362-365.

34. Gilpin, D. (correspondence); Pattison, S.; Wei, L.; Elborn, S.; Tunney, M. Molecular comparison of pseudomonas aeruginosa isolated from patients withCF and **non**-**CF bronchiectasis**. Pediatric Pulmonology, (September 2012) Vol. 47, Supp. SUPPL. 35, pp. 329.

35. Harrison, M.J. (correspondence); Murphy, D.M.; Henry, M.T.; Plant, B.J.;Kennedy, M.P. The clinical utility of a hand-held nasal nitric oxide electrochemicalanalyser to screen patients with **bronchiectasis** for Primary CiliaryDyskinesia (PCD) and Cystic Fibrosis (CF). Pediatric Pulmonology, (September 2012) Vol. 47, Supp. SUPPL. 35, pp. 310.

36. Boyton, Rosemary J. **Bronchiectasis**. Medicine, (May 2012) Vol. 40, No. 5, pp. 267-272.

37. Leighton, K.E. (correspondence); Stretton, R.J.; Fardon, T.C.; Schembri,S. Underlying causes of **bronchiectasis** identified in a specialist**non**-**CF bronchiectasis** service. Thorax, (December 2012) Vol. 67, Supp. SUPPL. 2, pp. A142-A143.

38. Ejiofor, S. (correspondence); Packer, G.; Mckinley, K.; Whitehouse, J. Outcomes of Pseudomonas Aeruginosa (PA) eradication in non -cysticfibrosis bronchiectasis . Forced Vital Capacity (FVC) and latent periodfrom growth to eradication are significant variables in eradication

success. Thorax, (December 2012) Vol. 67, Supp. SUPPL. 2, pp. A139-A141.

39. Coleman, M.C. (correspondence); Hennessy, C.; Bilton, D.; Wilson, R.;Loebinger, The short term variability of sputum microbiology in non –CF bronchiectasis . Thorax, (December 2012) Vol. 67, Supp. SUPPL. 2, pp. A139.

40. Baker, E.H. (correspondence); Kumar, N.; Sansom, B.; Nair, A.; Vlahos, I. Severity of bronchiectasis on high resolution ct scanning and its relationship to chronic bacterial colonisation. Thorax, (December 2012) Vol. 67, Supp. SUPPL. 2, pp. A138-A139.

41. Silvera, S.; Vignaux, O.; Szwarc, D. Peripherally-inserted central catheter for intravenous antibiotics inadult patients with cystic fibrosis or bronchiectasis . Journal of Cystic Fibrosis, (June 2012) Vol. 11, Supp. SUPPL. 1, pp. S131.

42. Sunny, S.S. (correspondence); Jary, H.; De Soyza, A. Osteoporosis in non -cystic fibrosis bronchiectasis (NCFBR) adults. Thorax, (December 2012) Vol. 67, Supp. SUPPL. 2, pp. A102-A103.

43. Bergin, D.A. (correspondence); Reeves, E.P.; Metha, A.; Amersi, P.; Ryan,D.; O'Neill, S.J.; McElvaney, N.G. Comparative analysis of inflammatory markers in cystic fibrosis and

non -cystic fibrosis bronchiectasis . Journal of Cystic Fibrosis, (June 2012) Vol. 11, Supp. SUPPL. 1, pp. S94.

44. Olveira Fuster, G. (correspondence); Dorado Galindo, A.; Olveira Fuster,C.; Espildora Hernandez, F.; Porras Perez, N.; Garcia Fuentes, E.; Gaspar,I.; Jimeno, R.; De La Cruz, J.L.; Murri, M. Oxidative stress biomarkers in cystic fibrosis and noncystic fibrosis bronchiectasis patients. Journal of Cystic Fibrosis, (June 2012) Vol. 11, Supp. SUPPL. 1, pp. S94.

45. Pattison, S. (correspondence); Wei, L.; Drain, M.; Tunney, M.; Elborn, J.S.; Gilpin, D.F. Molecular comparison of Pseudomonas aeruginosa isolated from patients withCF and non -CF bronchiectasis . Journal of Cystic Fibrosis, (June 2012) Vol. 11, Supp. SUPPL. 1, pp. S87.

46. Einarsson, G.G. (correspondence); Wei, L.; Tunney, M.; Elborn, J.S. Culture dependent and independent analysis of microbial community composition in the lungs of patients with cystic fibrosis and non -cystic fibrosis bronchiectasis . Journal of Cystic Fibrosis, (June 2012) Vol. 11, Supp. SUPPL. 1, pp. S83.

47. Wurzel, D. (correspondence); Marchant, J.M.; Brent Masters, I.; Chang, A.B. Cochrane systematic review-short courses of antibiotics for children and adults with bronchiectasis . Respirology, (April 2012) Vol. 17, Supp. SUPPL. 1, pp. 78.

48. Kapur, N. (correspondence); Masters, I.B.; Chang, A.B. Addressing major clinical gaps in paediatric non -cystic fibrosis (CF) bronchiectasis . Respirology, (April 2012) Vol. 17, Supp. SUPPL. 1, pp. 76.

49. Whitters, D. (correspondence); Stockley, R.A. Endotoxin specific IgG2 antibodies impair bacterial killing in non cystic fibrosis bronchiectasis patients colonised with pseudomonas

aeruginosa. Thorax, (December 2012) Vol. 67, Supp. SUPPL. 2, pp. A51.

50. Lake, A. Use of matrix-assisted laser desorption ionisation-time of flight(MALDI-TOF) mass spectrometry for identification of non-fermentative Gram-negative bacteria in cystic fibrosis and non -CF bronchiectasis . Journal of Cystic Fibrosis, (June 2012) Vol. 11, Supp. SUPPL. 1, pp. S45.
